# An Endophytic *Trichoderma* Strain Promotes Growth of Its Hosts and Defends Against Pathogen Attack

**DOI:** 10.3389/fpls.2020.573670

**Published:** 2020-12-03

**Authors:** Yu-Heng Tseng, Hamid Rouina, Karin Groten, Pijakala Rajani, Alexandra C. U. Furch, Michael Reichelt, Ian T. Baldwin, Karaba N. Nataraja, Ramanan Uma Shaanker, Ralf Oelmüller

**Affiliations:** ^1^Department of Plant Physiology, Matthias Schleiden Institute of Genetics, Bioinformatics and Molecular Botany, Friedrich-Schiller-University Jena, Jena, Germany; ^2^Department of Molecular Ecology, Max-Planck-Institute for Chemical Ecology, Jena, Germany; ^3^School of Ecology and Conservation, University of Agricultural Sciences, Gandhi Krishi Vigyana Kendra (GKVK), Bengaluru, India; ^4^Department of Crop Physiology, University of Agricultural Sciences, Gandhi Krishi Vigyana Kendra (GKVK), Bengaluru, India

**Keywords:** *Trichoderma*, plant beneficial endophyte, growth promotion, pathogen protection, hormone induction

## Abstract

Plants host numerous endophytic microbes which promote plant performance, in particular under stress. A new endophytic fungus was isolated from the leaves of a deciduous wood tree *Leucas aspera.* Morphological inspection and multilocus phylogeny identified the fungus as a new *Trichoderma* strain. If applied to *Arabidopsis thaliana* and *Nicotiana attenuata*, it mainly colonizes their roots and strongly promotes initial growth of the plants on soil. The fungus grows on high NaCl or mannitol concentrations, and shows predatory capability on the pathogenic fungus *Alternaria brassicicola*. Colonized *Arabidopsis* plants tolerate higher salt stress and show lower *A. brassicicola* spread in roots and shoots, while arbuscular mycorrhiza formation in *N. attenuata* is not affected by the *Trichoderma* strain. These beneficial features of the novel *Trichoderma* strain are important prerequisites for agricultural applications.

## Introduction

*Trichoderma* species are versatile filamentous ascomycetes which are found in nearly all environments. They live in soil, grow on wood as saprophytes, or feed on fungi, plants, animals and insects as parasites (Carsolio et al., 1994; Gautheret et al., 1995; Furukawa et al., 1998; El-Katatny et al., 2000; Rocha-Ramirez et al., 2002; Druzhinina et al., 2011; Li et al., 2013; Mukherjee et al., 2014; Li Destri Nicosia et al., 2015; Berini et al., 2016; Druzhinina and Kubicek, 2016; Rosmana et al., 2016; Karlsson et al., 2017). Various *Trichoderma* species were shown to protect plants against pathogenic fungi, such as *Rhizoctonia solani* (Grosch et al., 2007; Zhang and Zhuang, 2020). Therefore, they are commonly used as bio-control agents in agriculture, with more than 250 commercial *Trichoderma*-based bio-fungicides registered world-wide (Woo et al., 2014). Apart from being used as bio-fungicide, *Trichoderma* species also stimulate plant growth (Lee et al., 2016) and nutrient uptake under nutrient deficient conditions (Li et al., 2015), often in combination with better stress tolerance of crop plants (Studholme et al., 2013). Other species, such as *T. pleuroti* (CBS124387) and *T. pleuroticola* (CBS124383) cause green mold disease in oyster mushroom (*Pleurotus ostreatus*) farms (Park et al., 2006).

Most of the investigated *Trichoderma* species colonize either the root surface, or live as endophytes inside root tissues (Samolski et al., 2012; Ruano-Rosa et al., 2016). However, some species were also isolated from the aerial parts of the plants (Bailey and Melnick, 2013). In response, plants often activate defense mechanisms including the biosynthesis of the defense-related phytohormones salicylic acid (SA), jasmonic acid (JA), ethylene (ET) or abscisic acid (ABA) (Contreras-Cornejo et al., 2009; Hermosa et al., 2012; Sivakumaran et al., 2016; Checker et al., 2018). The phytohormones regulate two types of induced resistance in plants, namely, SA-dependent systemic acquired resistance (SAR) and JA/ET-dependent induced systemic resistance (ISR). The signaling events induced by *Trichoderma* species often result in elevated SA and JA levels in different parts of the plant (Martínez-Medina et al., 2013; Leonetti et al., 2017).

In this study, we wanted to find out if the novel endophytic *Trichoderma* strain isolated from the leaves of *Leucas aspera* also interacts with other plant species (*Arabidopsis*, *Nicotiana attenuata*) and has beneficial effects in terms of plant growth and alleviation of abiotic and biotic stress. We could show that, although the strain is phylogenetically related to mushroom-infecting *T. pleuroti* and *T. pleuroticola*, it efficiently colonizes the roots of the two plant species, strongly promotes their growth on soil during early development and protects them against systemic *A. brassicicola* spread, while mycorrhiza formation in *N. attenuata* appears not to be affected. We also evaluated if phytohormones might be involved in the plant-fungus interaction.

## Materials and Methods

### Growth Medium and Conditions for Seedlings

Seeds of wild-type *A. thaliana* (ecotype Columbia-0) were surface-sterilized for 8 min in sterilizing solution containing lauryl sarcosine (1%) and Clorix cleaner (23%). Surface-sterilized seeds were washed with sterilized water eight times and placed on Petri dishes with MS medium supplemented with 0.3% gelrite (Murashige and Skoog, 1962). After cold treatment at 4°C for 48–72 h, plates were incubated at 22°C under long day conditions (16 h light/8 h dark; 80 μmol m^–2^ s^–1^).

*Nicotiana attenuata* Torr. ex S. Watson seeds of the 31st generation of an inbred accession originally collected from southwestern Utah were used for all experiments mentioned for this species. Seeds were germinated after surface sterilization and treatment with liquid smoke (1:50 dilution; House of Herbs, Passaic, NY, United States) and 1 mM of gibberellic acid (GA_3_; Duchefa-Biochemie, The Netherlands) on agar plates containing Gamborg’s B5 medium as previously described in Krügel et al. (2002). Seeds were kept in a growth chamber under a day/night cycle of 16 h (26–28°C)/8 h (24–26°C).

### Growth of Fungi and Spore Collection

Based on our previous screen for plant growth-promoting fungi in a field station in India, the new *Trichoderma* strain was selected for detailed characterization. It was isolated from the leaves of *Leucas aspera* (Wild.) Link (family: *Lamiaceae*), a widely distributed medicinal plant reported for its antifungal, antioxidant, antimicrobial and cytotoxic activities (Prajapati et al., 2010; Rajani et al., 2020). The *Trichoderma* strain was grown on Petri dishes with Kaefer medium (KM) or Potato-Dextrose-Agar (PDA) medium, pH 6.5, at 23°C in the dark (Bains and Tewari, 1987; Hill and Käfer, 2001). *Alternaria brassicicola* was grown on Potato-Dextrose-Agar (PDA) medium, pH 6.5, 23°C in the dark (Bains and Tewari, 1987). We did not observe any difference of the fungal performance on the two media. Two additional pathogens, *Fusarium brachygibbosum* and *Alternaria* spp. isolate Utah 10, native to the natural habitat of *N. attenuata* isolated in a previous study (Luu et al., 2015), were grown on PDA medium at 26°C in the dark.

For spore collection, sterilized 0.01% Tween 20 solution was poured onto plates with fungi which were grown for less than 2 weeks. Spores were scratched from the agar surface and dispersed in 0.01% Tween 20. The resulting spore suspension was filtered through two layers of nylon membrane (75 μm pore size, Sefar AG, Switzerland), pelleted and washed with sterile distilled water. The *A. brassicicola* spore concentration was determined in a hemocytometer, while the *Trichoderma* spore concentration was determined by O.D._600 nm_ measurements using a spectrometer (BioSpectrometer^®^ basic, Eppendorf, Germany).

For co-cultivation experiments with *N. attenuata*, the *Trichoderma* strain was cultivated on PDA plates. Spores of 7–14 day-old cultures were dislodged from the surface with sterile distilled water containing 0.01% Triton X-100. The resulting solution was diluted with distilled water to an O.D._600 nm_ of 0.250–0.350.

### Co-cultivation of *A. thaliana* and *N. attenuata* With *Trichoderma*

Co-cultivation of *A. thaliana* and fungi was performed according to Johnson et al. (2011) with modifications. A plug (5 mm diameter) from a KM plate containing the fungus or a control plug without the fungus was put on a fresh plate with solid plant nutrient medium (PNM), which contained a layer of a nylon membrane (pore size 75 μm) on the agar surface. The plates were incubated at 23°C for 7 days. Unless specified, four 10 day-old *A. thaliana* seedlings of equal size were transferred to the plates. They were incubated at 22°C under long day conditions (16 h light/8 h dark; 80 μmol m^–2^ s^–1^).

For co-cultivation of *A. thaliana* with *Trichoderma* on soil, 1 kg of soil was suspended in 5 L of distilled water overnight. The liquid was removed by filtration and the soil was autoclaved twice. 200 g of the soil was transferred to magenta boxes for co-cultivation assays. The soil was inoculated with a 5 mm plug of KM medium with or without *Trichoderma* 3 cm below the soil surface in the center of the box. 10 day-old *Arabidopsis* seedlings were transferred to the soil, and the boxes were kept at 22°C under long day conditions (16 h light/8 h dark; 80 μmol m^–2^ s^–1^) for 4 weeks.

Co-cultivation of *N. attenuata* with *Trichoderma* was performed in Petri dishes and on soil. For experiments in Petri dishes, sterilized seeds treated with liquid smoke (1:50 dilution; House of Herbs, Passaic, NY, United States) and GA (1 mM GA_3_; Duchefa-Biochemie, The Netherlands) were incubated for 1 h with a highly diluted spore and hyphae solution before transfer to GB5 medium (see Santhanam et al., 2019 for experimental details). In a second set-up, liquid smoke-and GA-treated seeds were germinated on GB5 medium for 7 days before they were transferred in a circle with 10 seedlings to a new plate. Immediately after transfer roots either received 10 μL sterile distilled water or the same amount of spore solution (O.D._600 nm_ = 0.2653). One day later an Agar plug of *Alternaria* spp. Utah 10 was placed in the middle of the plate. Inoculated seedlings were kept at 26°C and 14 h light and 10 h dark cycle for 16 days.

For pot experiments on soil, *Trichoderma* treated seedlings and controls were transferred to pots and cultivated in a Snijders Chamber with a 16 h light/8 h dark cycle at 65% relative humidity.

To study the effect of *Trichoderma* strain on arbuscular mycorrhizal fungi (AMF), *N. attenuata* seedlings were transferred to Teku pots with sand 10 days after germination, and transferred to 10% of the commercial inoculum (BiomycVital, which contains AMF spores and tiny pieces of roots/hyphae in expanded clay)^1^ after another 12 days. Upon transfer, half of the plants received a *Trichoderma* spore solution, while control plants received the same amount of distilled water. Plants were watered with hydroponics solution containing 0.05 mM P. Roots were collected for further analysis 8 weeks after transfer.

### Nucleic Acid Isolation, Primers, and PCR and Sequencing

*Arabidopsis* root and fungal tissue were ground in liquid nitrogen, and DNA extraction was performed according to Doyle (1990). RNA from AMF-colonized *N. attenuata* roots was extracted with the LiCl method according to Kistner and Matamoros (2005). RNA samples were treated with DNAse removal kit (Ambion, Thermo Fisher Scientific, Germany) according to the manufacturer’s instructions and reverse transcribed with Superscript II (Invitrogen, Thermo Fisher Scientific, Germany) and Oligo-dT.

The primer pairs for amplifying the *TEF1* (translation elongation factor 1-alpha) and *RPB2* (RNA polymerase II subunit 2) genes from *Trichoderma* are: TEF1-F: 5′-CATCGAGAAGTTCGAGAAGG-3′; TEF1-R: 5′-AACTTGCAG GCAATGTGG-3′; RPB2-F: 5′-TGGGGWGAYCARAARAAGG-3′; RPB2-R: 5′-CATRATGACSGAATCTTCCTGGT-3′. Each 20 μL PCR reaction contains 2 μL of 10× DreamTaq Buffer (Thermo Fisher Scientific, Germany), 0.2 mM dNTP, 1.0 μM forward/reverse primer, 100 ng genomic DNA template and 1U of DreamTaq DNA Polymerase (Thermo Fisher Scientific, Germany). The reaction was performed in a thermal cycler (Applied Biosystems SimpliAmp Thermal Cycler, Thermo Fischer Scientific, Germany). The initial denaturation step was set at 95°C for 3 min, followed by 30 cycles of denaturation at 95°C for 30 s, annealing at 55°C (*TEF1*) or 62°C (*RPB2*) for 30 s, and extension at 72°C for 30 s. The final extension step was set at 72°C for 10 min. The PCR products from at least two independent PCR runs were purified by NucleoSpin Gel and PCR Clean-up kit (Macherey-Nagel, Germany). Purified PCR products were sent to Eurofins Genomics for Sanger sequencing. Consensus sequence of *TEF1* and *RPB2* was deposited to Genbank with accession numbers MT591352 and MT602550, respectively.

To quantify the colonization of *Trichoderma* under various salt concentrations, *Trichoderma TEF1* and *A. thaliana RPS* (AT1G34030) were detected by qPCR with the following primers: TEF-qF: 5′-TCAAGTCCGTTGAGATGCAC-3′; TEF-qR: 5′-CGTTCTTGACGTTGAAACCA-3′; RPS-qF: 5′- GT CTCCAATGCCCTTGACAT-3′; RPS-qR: 5′- TCTTTCCTCTGCGACCAGTT-3′.

For qPCR analysis of AMF colonization of *N. attenuata* roots, qPCR reactions were performed on Mx3005P qPCR system (Stratagene, Santa Clara, CA, United States) with Takyon Sybr Green No ROX kit (Eurogentec, Belgium). Primers for *NaRAM1*, *NaPT4*, and *Rhizophagus irregularis tubulin* are from Wang et al. (2018a). Primers for *NaEF1* are from Wang et al. (2018b). Primers for *RPB2* of *Trichoderma* are: RPB2-qF: 5′-AGACGTCCATGATCTGCATGAC-3′; RPB2-qR: 5′-TGTCTTGGTCTTGAGTCGCTTG-3′

The genes for *A. thaliana Ubiquitin 5*, *N. attenuata Elongation Factor 1 alpha* (*NaEF1*, Wang et al., 2018b) and *A. brassicicola Cutinase 1* were used to monitor *A. brassicicola* infection in root tissue. The primer pairs for qPCR analysis are: AtUBQ5-qF: 5′-GACGCTTCATCTCGTCC-3′; AtUBQ5-qR: 5′-GTAAACGTAGGTGAGTCCA-3′; AbCUT1-qF: 5′-GACCGAGGAAGCTCAGATGC-3′; AbCUT1-qR: 5′-GCCTGGGATCTTGGAATGC-3′.

### Multilocus Phylogenetic Analysis

The nucleotide sequences of *TEF1* and *RPB2* from 55 *Trichoderma* species and an outgroup species, *Nectria eustromatica*, were retrieved from the NCBI Nucleotide public database. The *TEF1* and *RPB2* sequences of the new *Trichoderma* strain were obtained from the PCR products. *TEF1* and *RPB2* genes from the same species were concatenated for combined analysis. In total, 56 concatenated sequences were subjected to alignment using MAFFT v7 online at https://mafft.cbrc.jp/alignment/server (Katoh et al., 2019), with G-INS-i parameters and a scoring matrix of “1PAM/κ = 2” for nucleotide sequences. The resulting alignment was inspected and selected for conserved blocks using Gblocks version 0.91b (Castresana, 2000).

Maximum likelihood analysis was conducted using RaxML-NG v.0.9.0 through web service at https://raxml-ng.vital-it.ch (Kozlov et al., 2019). Using the GTR+FO+G4m model, 2000 distinct ML tree were searched and bootstrapped with 100 replicates. For maximum parsimony analysis, PAUP 4.0a166 was utilized (Swofford, 2002). Heuristic search of 100 replicates was performed with random addition of sequence, and tree bisection-reconnection (TBR) as the branch-swapping algorithm (steepest decent and MulTrees option not in effect). All characters were weighted equally, and gaps were treated as missing character. Bootstrap of 1,000 replicates was undertaken with Maxtrees set as 5,000.

The Bayesian analysis was conducted using MrBayes v3.2.7a (Huelsenbeck and Ronquist, 2001; Ronquist and Huelsenbeck, 2003). The evolutionary model was set to the general time-reversible model (GTR; Tavare, 1986), and the nucleotide variation rate set to inverse gamma distribution (Yang, 1993). Two simultaneous and independent Markov chain Monte Carlo (mcmc) analyses was run to generate 1 million generations each, while they were sampled for every 10 generations to determine the posterior probability (Geyer, 1991). From the resulting 100,000 sampled trees, the first 25% of them were discarded, and the remaining 75,000 trees were summarized to produce the consensus tree.

The Maximum likelihood boostrap proportions (MLBP), Maximum parsimony bootstrap proportions (MPBP), as well as the Bayesian inference posterior probability (BIPP) from each analysis were combined to the phylogenetic tree from the RAxML analysis using TreeGraph2.15.0–887 beta (Stöver and Müller, 2010). The final tree was created with FigTree v1.4.4 (Rambaut, 2018). The accession numbers of the individual genes are provided in Supplementary Table 1.

### Histological Staining and Microscopy

Roots of *A. thaliana* co-cultivated with the *Trichoderma* strain for 2 or 7 days were collected and immersed in Wheat Germ Agglutinin, Alexa Fluor^TM^ 488 Conjugate (Thermo Fisher Scientific, Germany) for 10 min in dark. Immersed samples were taken out from the staining solution and placed on a glass slide. Water was applied to the slide to wash away excess staining solution and the slide was covered with a cover slip for microscopic inspection with Axio Imager.M2 (Zeiss Microscopy GmbH, Germany). The bright field and fluorescent images were recorded with a monochromatic camera Axiocam 503 mono (Zeiss Microscopy GmbH, Germany). Digital images were processed with the ZEN software (Zeiss Microscopy GmbH, Germany).

For confocal imaging of root colonization, *A. thaliana* roots co-cultivated with *Trichoderma* for 2 days were stained with Wheat Germ Agglutinin, Alexa Fluor^TM^488 Conjugate and RH414 [N-(3-Triethylammoniumpropyl)-4-(4-(4-(Diethylamino)phenyl)Butadienyl)Pyridinium Dibromide; Thermo Fischer Scientific, Germany] with the method described above. Samples were imaged using an LSM 880 microscope (Zeiss Microscopy GmbH, Germany) with the 488 nm laser line of an argon multiline laser (11.5 mW). Images were taken with a 40× objective (Plan-Apochromat 40×/0.8). Lambda stacks were created using the 32 channel GaAsP detector followed by Linear Unmixing with the ZEN software. Z-stacks were taken from specific areas of the sample and Maximum Intensity Projections were produced with the ZEN software.

### Phytohormone Analyses by LC-MS/MS

Sixteen seedlings from control and co-cultured plates were harvested and separated into root and shoot samples. Mycelium of the *Trichoderma* strain grown on KM plates was harvested for phytohormone analysis.

Fifty to one hundred thirty milligrams of fresh tissue were extracted and homogenized in 1.5 mL methanol containing 60 ng D4-SA (Santa Cruz Biotechnology, United States), 60 ng D6-JA (HPC Standards GmbH, Germany), 60 ng D6-ABA (Santa Cruz Biotechnology, United States), 12 ng D6-JA-Ile (HPC Standards GmbH), and D5-indole-acetic acid (D5-IAA, OlChemIm s.r.o., Olomouc, Czech Republic) as internal standards. Samples were agitated on a horizontal shaker at room temperature for 10 min. The homogenate was mixed for 30 min and centrifuged at 13,000 rpm for 20 min at 4°C and the supernatant was collected. The homogenate was re-extracted with 500 μL methanol, mixed and centrifuged and the supernatants were pooled. The combined extracts were evaporated under reduced pressure at 30°C and dissolved in 500 μL methanol.

Phytohormone analysis was performed by LC-MS/MS as in Heyer et al. (2018) on an Agilent 1260 series HPLC system (Agilent Technologies) with the modification that a tandem mass spectrometer QTRAP 6500 (SCIEX, Darmstadt, Germany) was used. Since we observed that both the D6-labeled JA and D6-labeled JA-Ile standards (HPC Standards GmbH, Cunnersdorf, Germany) contained 40% of the corresponding D5-labeled compounds, the sum of the peak areas of the D5- and D6- compounds was used for quantification. Details of the instrument parameters and response factors for quantification can be found in Supplementary Table 2.

Indole-acetic acid was quantified using the same LC-MS/MS system with the same chromatographic conditions but with positive mode ionization with an ion spray voltage at 5,500 eV. Multiple reaction monitoring (MRM) was used to monitor analyte parent ion → product ion fragmentations as follows: m/z 176 →130 [collision energy (CE) 19 V; declustering potential (DP) 31 V] for indole-acetic acid (IAA); m/z 181 →133 + m/z 181 →134 + m/z 181 →135 (CE 19 V; DP 31 V) for D5-indole-acetic acid.

### Quantification of Mycelial Growth, AMF Colonization, and 11-Carboxyblumenol Levels

Plates with mycelia were scanned with an Epson scanner (Perfection V600 Photo, Epson, Germany), and the files imported into ImageJ (Schindelin et al., 2012). Mycelial coverage on each plate was delineated using a free-hand selection tool and measured with the built-in “Measure” function.

AMF colonization was determined by the “magnified intersections method” described in detail by McGonigle et al. (1990). In brief, roots were cut in about 1 cm pieces and we counted the fungal structures of 150 intersections per sample after staining with Trypan Blue.

For determination of AMF colonization marker 11-carboxyblumenol, three leaf disks per AMF inoculated of the first and second stem leaf were harvested 6 and 8 weeks after AMF inoculation. 11-Carboxyblumenol levels were determined as markers of arbuscule colonization and quantified following the protocol of Wang et al. (2018a).

### Statistical Tests

Statistical tests were performed using R studio version 1.1.463 with R version 3.4.4. Figures were plotted using Python 3.7.4 and arranged with LibreOffice Draw 5.1.6.2.

## Results

### Morphological and Phylogenetic Analysis of the New *Trichoderma* Strain

The *Trichoderma* strain was isolated from leaves of *Leucas aspera* (Wild.) Link. We selected this strain for further analysis because we observed in preliminary field experiments that it promotes growth of several crop species. Its morphology shows typical characteristics of *Trichoderma* species of the *harzianum* clade. On KM plates, the hyphae cover the entire Petri dish from a single plug (5 mm in diameter) in 3–4 days (Supplementary Figure 1A). During the first 3 days after transfer to a new plate, the hyphae extended and formed conidiophores at the tip of hyphal branches. The conidia grew, replicated, and aggregated at the tip of a conidiophore (Supplementary Figure 1B). After 7 days, mature conidia developed as sphere-like structure composed of numerous individual conidia (Supplementary Figure 1C). The hyphal cell shrank after the conidia were fully developed. This allowed them to detach from the hyphae (Supplementary Figure 1D). The fully matured conidia displayed a green color (Supplementary Figure 1E).

**FIGURE 1:**
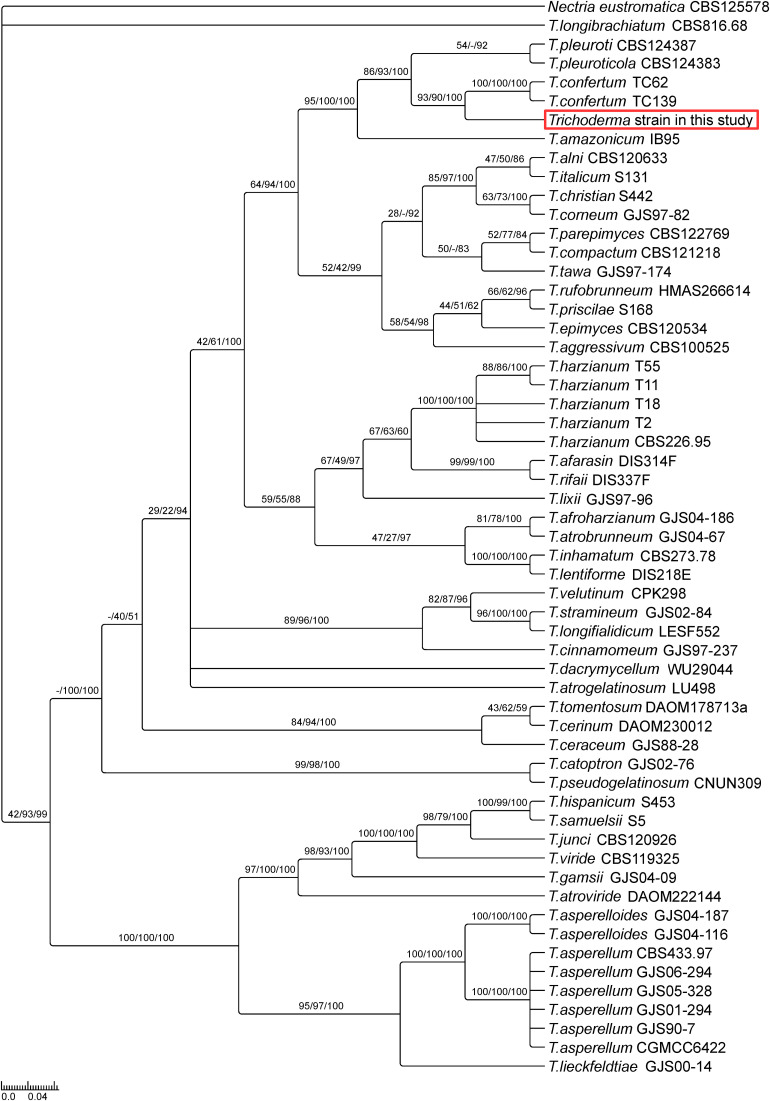
Phylogeny of selected *Trichoderma* species suggests a new *Trichoderma* strain closely related to *T. confertum*. The phylogenetic tree is based on combined analysis of *TEF1* and *RPB2* genes using Bayesian inference of phylogeny. Maximum likelihood bootstrap values (MLBP, left), maximum parsimony bootstrap values (MPBP, center) and Bayesian inference posterior probabilities (BIPP, right) are shown at each node. *Nectria eustromatica* was used as outgroup.

Phylogenetic analysis based on Maximum likelihood, Maximum Parsimony and Bayesian Inference of phylogeny uncovered that the isolated *Trichoderma* strain belongs to the *harzianum* clade (Figure 1), closely related to *T. confertum* TC62 and *T. confertum* TC139, two strains recently isolated from the soil 2,000 m above sea level in Tibet (Chen and Zhuang, 2017). The multilocus sequence analysis also indicated that the strain is closely related to *T. pleuroti* and *T. pleuroticola*, but less compared to *T. confertum.* In summary, according to the three different analysis methods, the isolated fungus is most probably a new *Trichoderma* strain closely related to *T. confertum*.

### The New *Trichoderma* Strain Colonizes *Arabidopsis* and *Nicotiana* Roots and Promotes Plant Growth

**FIGURE 2:**
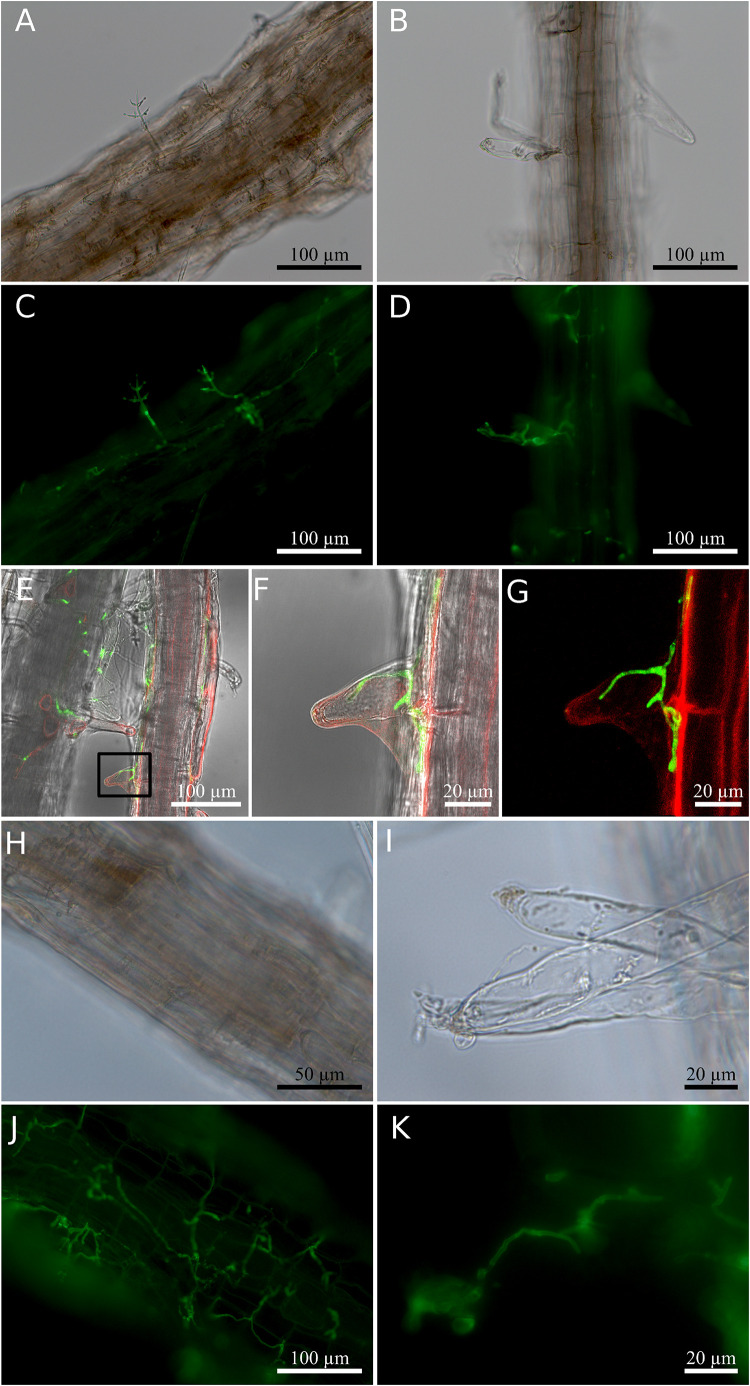
Root colonization of *Arabidopsis thaliana* by *Trichoderma*. **(A–D)** Co-cultivation for 2 days. **(A,B)** Bright field; **(C,D)** fluorescence of fungal stain. **(E)** Confocal images of hyphae inside root hair observed 2 days after co-cultivation. **(F)** Magnified view of the region enclosed by the small box in **(E)**. **(G)** Fluorescence signal indicating hyphae (green) and plant cell plasma membrane (red) in **(F)**. **(H–K)** Co-cultivation for 7 days. **(H,I)** Bright field; **(J,K)** fluorescence of fungal stain. Colonized root tissues were stained with WGA Alexa Fluor^TM^ 488 conjugate to detect the presence of the fungus, and RH414 was used to visualize the plant plasma membrane. The image shown for the confocal microscopy was chosen from three individual roots of three biological replicates.

To characterize the endophytic lifestyle of the new strain, and to check which organ of the plant can be colonized by the fungus, it was co-cultivated with the model species, *Arabidopsis thaliana*. Two days after co-cultivation, hyphae were already detectable on the surface of the roots (Figures 2A,C). Light and confocal microscopy showed that hyphae also invaded into the root hair (Figures 2B,D–G and Supplementary Movie 1). Seven days after co-cultivation, the *Arabidopsis* roots were highly colonized, and conidiophores were found at the tip of the root hair, although not every root hair contained hyphae or conidiophore (Figures 2H–K). Close inspections revealed that the conidiophores derived from the hyphae in the root hairs. Under these co-cultivation conditions without stress, the aerial parts of the plant were not colonized.

**FIGURE 3:**
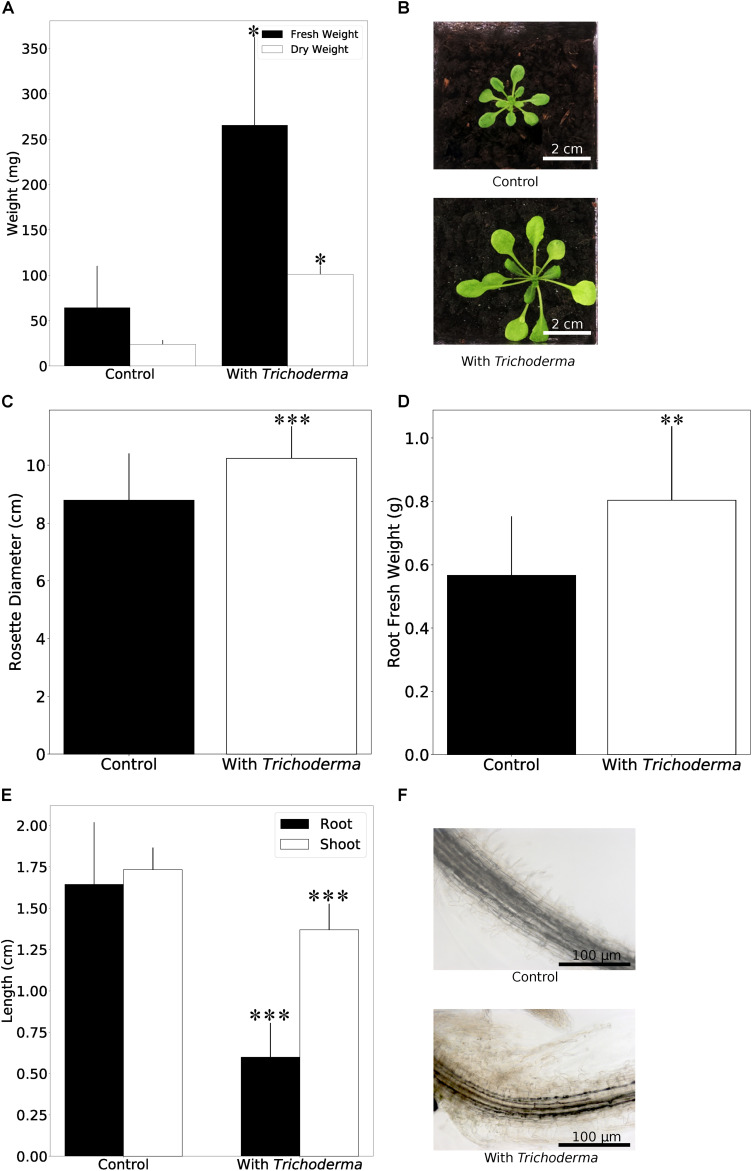
Plant growth performance is influenced by *Trichoderma* colonization. **(A)** Fresh and dry weights of *A. thaliana* grown on soil with or without *Trichoderma* after 4 weeks. Error bars represent SEs from three independent biological replicates, each with four seedlings. Statistical significance was determined by Welch Two Sample *t*-test (^∗^*P* < 0.05). **(B)** Growth promotion on *A. thaliana* on soil after 4 weeks. **(C,D)** Rosette diameter **(C)** and root fresh weight **(D)** of *N. attenuata* inoculated with or without *Trichoderma* on soil after 4 weeks. Error bars represent SDs from 39 independent biological replicates for shoots and 18 replicates for roots. Statistical significance was determined by Welch Two Sample *t*-test (^∗∗^*P* < 0.01; ^∗∗∗^*P* < 0.001). **(E)** Shoot and root lengths of *N. attenuata* 12 days after co-cultivation with *Trichoderma* (spore solution O.D._60__0 n__m_ = 0.0135) or without *Trichoderma* on Petri dishes. Error bars represent SDs from 10 biological replicates. Statistical significance was determined by Welch Two Sample *t*-test (^∗∗∗^*P* < 0.001). **(F)** Microscopy of *N. attenuata* roots 12 days on Petri dishes with or without *Trichoderma*.

When *Arabidopsis* plants were co-cultivated on soil in the greenhouse, the germination rate and the performance of the young seedlings were not affected by the fungus. However, we observed a strong initial growth-promoting effect of the fungus on 4-week old *Arabidopsis* plants, since colonized plants were almost twice as large as the uncolonized controls (Figures 3A,B). Root colonization by the *Trichoderma* strain was confirmed by microscopy (Supplementary Figure 2). During later stages, the growth difference between colonized and uncolonized plants became less and during flowering time, the growth-stimulating effect of the fungus was barely visible. The number and size of seeds was not significantly different for plants grown with or without the fungus (data not shown). This indicates that the fungus promotes plant growth during early stages of development.

Growth promotion was also observed for the model plant *N. attenuata* (cf. also below). We observed the same colonization efficiencies as described above for *Arabidopsis* seedlings. Also, the germination rates were similar for inoculated and non-inoculated seeds, and all seedlings were healthy. Similar to *Arabidopsis*, we observed a stimulatory effect of the fungus on *N. attenuata* growth after 4 weeks on soil, when both the rosette diameter and root biomass were larger (Figures 3C,D). Comparable to *Arabidopsis*, the growth-stimulating effect disappeared during later stages of development. However, we observed a clear difference in the response of the two hosts on agar plates during early seedling’s development, where root and shoot development can be monitored in more details. With fungal inoculation, the shoots and roots of 12-day-old *Arabidopsis* seedlings were bigger in the presence of the fungus (see Figure 5), while 12-day-old colonized *N. attenuata* had significantly shorter shoots and roots than the uncolonized controls (Figure 3E). We also observed more root hairs beneath the root-shoot junction, where roots are in contact with the fungus (Figure 3F). These effects were not observed for the roots of *Arabidopsis* seedlings. In conclusion, the fungus has different effects on the early development of the seedlings on agar medium.

### The New *Trichoderma* Strain Is Tolerant Against 100 mM Salt and Mild Salt Conditions Promote the Interaction With the Host on Synthetic Medium

**FIGURE 4:**
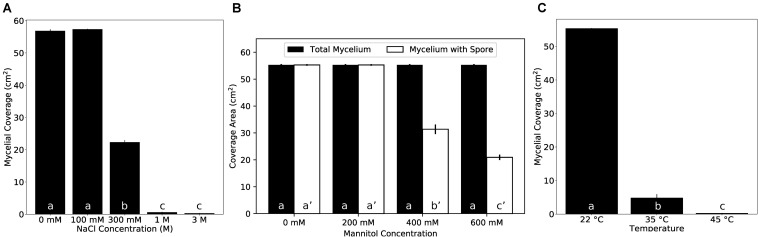
Growth of the *Trichoderma* strain under various conditions on KM plates. **(A)** Fungal growth on different NaCl concentrations. **(B)** Fungal growth on different mannitol concentrations. **(C)** Fungal growth at different temperatures. Error bars represent SEs from three independent biological replicates. Statistical significance was determined by Tukey’s HSD test with *P* < 0.05, and is indicated by different lower-case letters.

Plant growth promoting fungi and bacteria often also improve the stress tolerance of plants (Qin et al., 2016). Therefore, we first tested if the fungus itself is tolerant against salt and mannitol (osmotic) stress. Fungal growth was not altered on 100 mM NaCl compared to control plates without salt. At 300 mM NaCl, the mycelial growth was reduced by about 50%. At 1 M NaCl, only slowly growing mycelia could be detected after 10 days, and no growth was detectable on 3 M NaCl (Figure 4A and Supplementary Figures 3A–G). Increasing mannitol concentrations did not inhibit mycelial growth, although the production of conidia was reduced on media with >400 mM mannitol (Figure 4B and Supplementary Figures 3H–K). Also high temperature strongly impaired growth of the fungus (Figure 4C and Supplementary Figures 3L–N).

**FIGURE 5:**
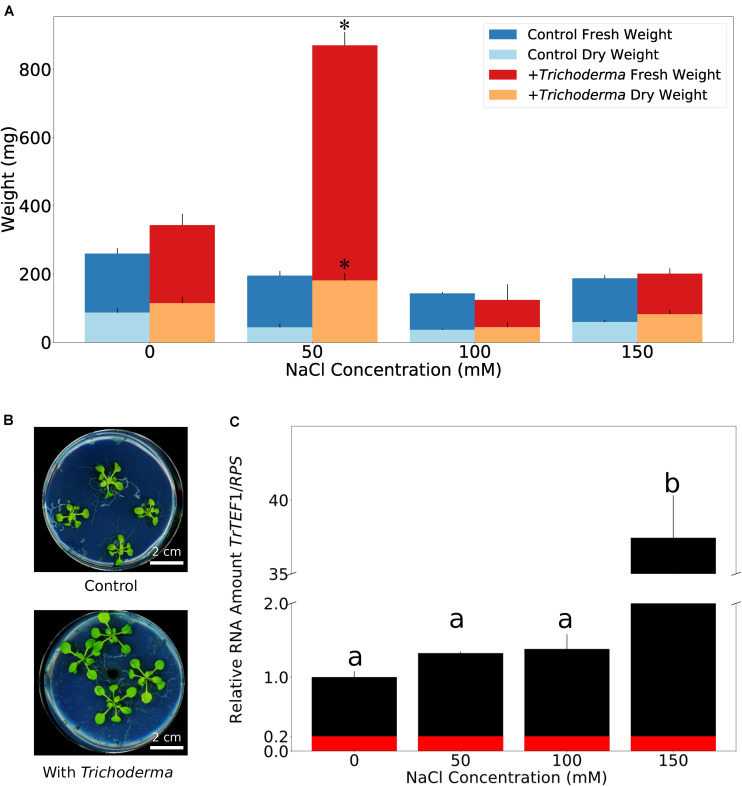
Mild salt condition optimizes fungal colonization and plant growth performance on synthetic medium. **(A)** Fresh and dry weights of *A. thaliana* co-cultivated with or without *Trichoderma* on PNM medium with 0–150 mM NaCl for 5 days. Error bars represent SEs from three independent biological replicates, each with four seedlings. Statistical significance was determined by 2-Way ANOVA. *Post-hoc* analysis between all groups was further carried out by Tukey HSD test with *P* < 0.05, and is shown in Supplementary Table 3. Asterisks indicate significant difference in fresh/dry weight of seedlings grown on 50 mM NaCl with or without *Trichoderma*. **(B)** Growth phenotype of *A. thaliana* on PNM medium with 50 mM NaCl 5 days after co-cultivation with or without *Trichoderma*. **(C)** Quantification of *Trichoderma* root colonization on *A. thaliana* on PNM medium with 0–150 mM NaCl by qPCR. *TrTEF1*: *Trichoderma TEF1*; *RPS*: *A. thaliana* ribosomal protein S13/S18 family. Error bars represent SEs from three independent biological replicates, each with four seedlings. Statistical significance was determined by Tukey’s HSD test with *P* < 0.05, and is indicated by different lower-case letters. Red color represents average background value referred from samples without *Trichoderma*.

The ability of the fungus to survive 1 M NaCl intrigued us to find out if salt influences the growth stimulating effect of its host. Unlike on soil, when seedlings were grown on solid PNM medium without addition of NaCl for 5 days, we observed only a small increase in growth and biomass production of the *Arabidopsis* seedlings in the presence of the fungus. However, application of 50 mM NaCl to the medium strongly promoted growth and biomass production (Figures 5A,B; supported by *post-hoc* analysis shown in Supplementary Table 3). This was accompanied by a stronger root colonization (Figure 5C). In particular, the lateral roots of the host were massively colonized and the fungus produced large amounts of conidiospores, compared to those on medium without NaCl. On 100 mM and higher NaCl concentrations, the growth of the uncolonized plants was gradually reduced, and growth of *Trichoderma*-colonized roots was not stimulated any more (Figure 5A and Supplementary Figure 4). Closer inspections uncovered that roots were even more colonized, and the hyphae also appeared on the surface of the areal parts. They were not only detectable at and around the hypocotyl (Supplementary Figure 4) but also on the leaf surface (data not shown). In summary, apparently, the fungus colonizes preferentially the roots. Low NaCl concentrations promoted root colonization and stimulated plant growth, while higher salt concentrations forced the fungus to invade the aerial parts which was associated with a loss of the benefits to the host.

### The New *Trichoderma* Strain Inhibits Growth of *Alternaria* and Protects *Arabidopsis* and *Nicotiana* Against *Alternaria* Infection

**FIGURE 6:**
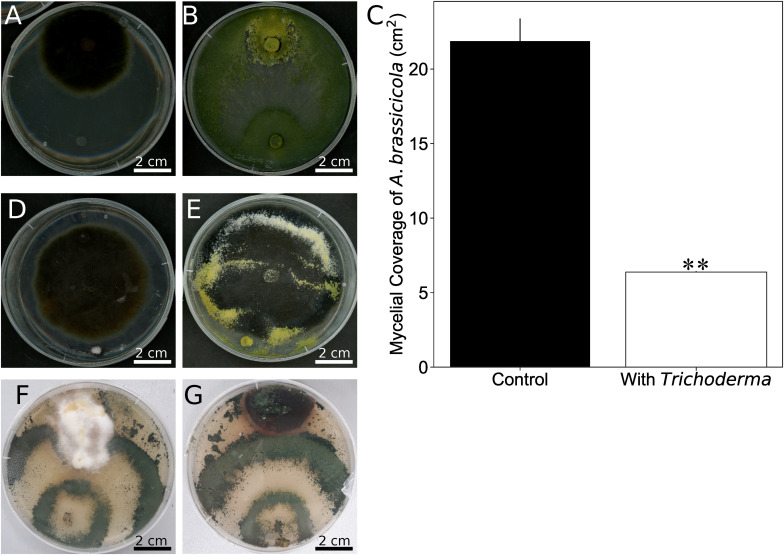
*Trichoderma* inhibits growth of *Alternaria brassicicola*. **(A,B)** A plug of *A. brassicicola* was applied to the upper side of the PDA plate. A plug of KM agar **(A)** or the *Trichoderma* strain **(B)** was put to the lower side of the plate before co-cultivation for 8 days. **(C)** Quantification of *A. brassicicola* mycelial coverage from **(A,B)**. Error bars represent SDs from three independent biological replicates. Statistical significance was determined by Welch Two Sample *t*-test (^∗∗^*P* < 0.01). **(D)** An agar plug with *Trichoderma* was placed on a PDA plate which contained 7-day old *A. brassicicola* culture. **(E)** Seven days after the plug with *Trichoderma* was placed in **(D)**. **(F,G)** Co-cultivation of *Trichoderma* with the native *N. attenuata* pathogens *Fusarium brachygibbosum* **(F)** or *Alternaria* spp. strain Utah 10 **(G)**.

One of the prominent traits of *Trichoderma* species in the *harzianum* clade is their potential to act as bio-control agent. After 8 days of co-cultivation of *Alternaria brassicicola* with the *Trichoderma* strain on PDA plates, the mycelial coverage of *A. brassicicola* was reduced by 73% and *Trichoderma* hyphae grew on top of the *A. brassicicola* mycelial lawn (Figures 6A–C). To rule out that faster growth of *Trichoderma* restricts *A. brassicicola* growth, *Trichoderma* was added to an agar plate with a 7-day old *A. brassicicola* culture (Figure 6D). After additional 7 days of co-cultivation, *Trichoderma* hyphae and spores were again observed on top of the *A. brassicicola* mycelial lawn (Figure 6E). This supports active predation of *A. brassicicola* by the new *Trichoderma s*train (Druzhinina et al., 2018).

We further tested *Fusarium brachygibbosum and Alternaria* spp. *Utah isolate 10*, two fungal species previously characterized as a native pathogen for *N. attenuta* (Luu et al., 2015), and co-cultivated them with *Trichoderma*. Growth of *Trichoderma* was much faster than that of the two other species, but *F. brachygibbosum* clearly stopped further growth of *Trichoderma* when hyphae of the two fungi met, while *Alternaria* spp. was overgrown by *Trichoderma* after 3½ weeks of co-cultivation (Figures 6F,G).

**FIGURE 7:**
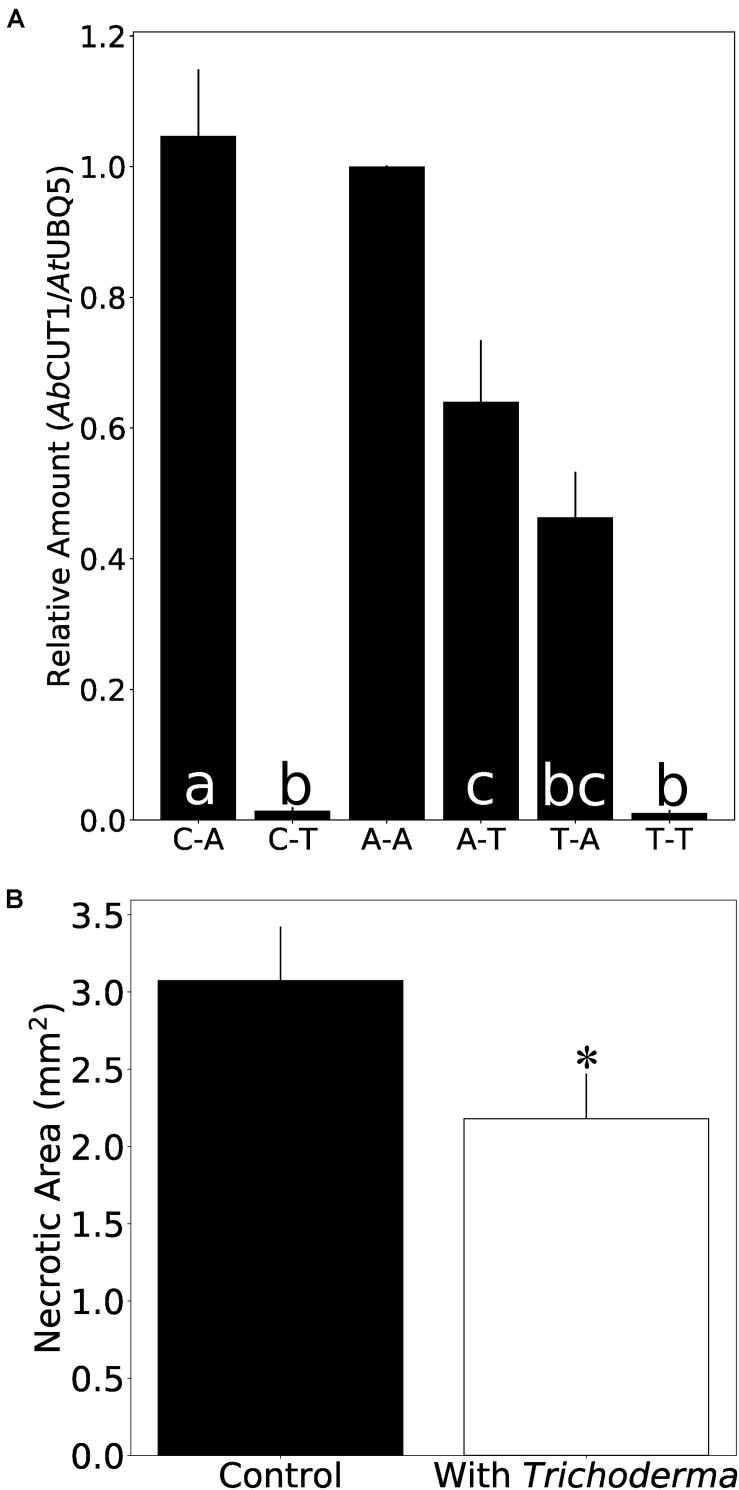
*Trichoderma* protects *Arabidopsis* seedlings against *A. brassicicola* infection. **(A)** Amount of *A. brassicicola CUT1* DNA relative to *A. thaliana UBQ5* DNA in root tissue. C-A: co-cultivation with control plug for 7 days, then co-cultivation with *A. brassicicola* for another 7 days. C-T: co-cultivation with control plug for 7 days, then co-cultivation with *Trichoderma* for another 7 days. A-A: co-cultivation with *A. brassicicola* for 7 days, then co-cultivation with *A. brassicicola* for another 7 days. A-T: co-cultivation with *A. brassicicola* for 7 days, then co-cultivation with *Trichoderma* for another 7 days. T-A: co-cultivation with *Trichoderma* for 7 days, then co-cultivation with *A. brassicicola* for another 7 days. T-T: co-cultivation with *Trichoderma* for 7 days, then co-cultivation with *Trichoderma* for another 7 days. Values from qPCR experiment were normalized to A-A. Error bars represent SEs from three independent biological replicates, each with 6–9 seedlings. Statistical significance was determined by Tukey’s HSD test with *P* < 0.05, and is indicated by different lower-case letters. **(B)** Necrosis area on leaflets infected by *A. brassicicola.* Error bars represent SEs from 35 independent biological replicates. Statistical significance was determined by Welch Two Sample *t*-test (^∗^*P* < 0.05).

To test if the *Trichoderma* strain also protects plants from *Alternaria* infection, *Arabidopsis* seedlings were first exposed to *A. brassicicola* (A) or *Trichoderma* (T) or were mock-treated (C) and then transferred to plates with either *A. brassicicola* or *Trichoderma* for additional 7 days. As expected, the highest amount of *A. brassicicola* DNA was detected in seedlings which were exposed to *A. brassicicola* only (Figure 7A). Roots which were exposed to *Trichoderma* either before or after *A. brassicicola* treatment (A-T) contained less DNA of the fungal pathogen. Furthermore, the seedlings were better protected against *A. brassicicola* when they were already colonized by *Trichoderma* before pathogen infection (Figure 7A and Supplementary Figure 5A). Similar results were observed for *N. attenuata* and *Alternaria* (Supplementary Figure 5B). This demonstrates that the *Trichoderma* strain restricts growth of the pathogen in roots of its host plant.

To investigate whether *Trichoderma* also protects the leaves against *A. brassicicola* infection, 500 colony forming units (CFU) of an *A. brassicicola* spore suspension were applied to the leaves of *Arabidopsis* seedlings which were either co-cultivated with the symbiont or mock-treated for 7 days. Four days later, the necrotic zone on the leaves of co-cultivated plants was significantly smaller compared to the non-colonized controls (Figure 7B). Taken together, *Trichoderma* restricts spread of *Alternaria* in both roots and shoots.

### Mycorrhiza Formation Is Not Affected by the New *Trichoderma* Strain in *N. attenuata*

**FIGURE 8:**
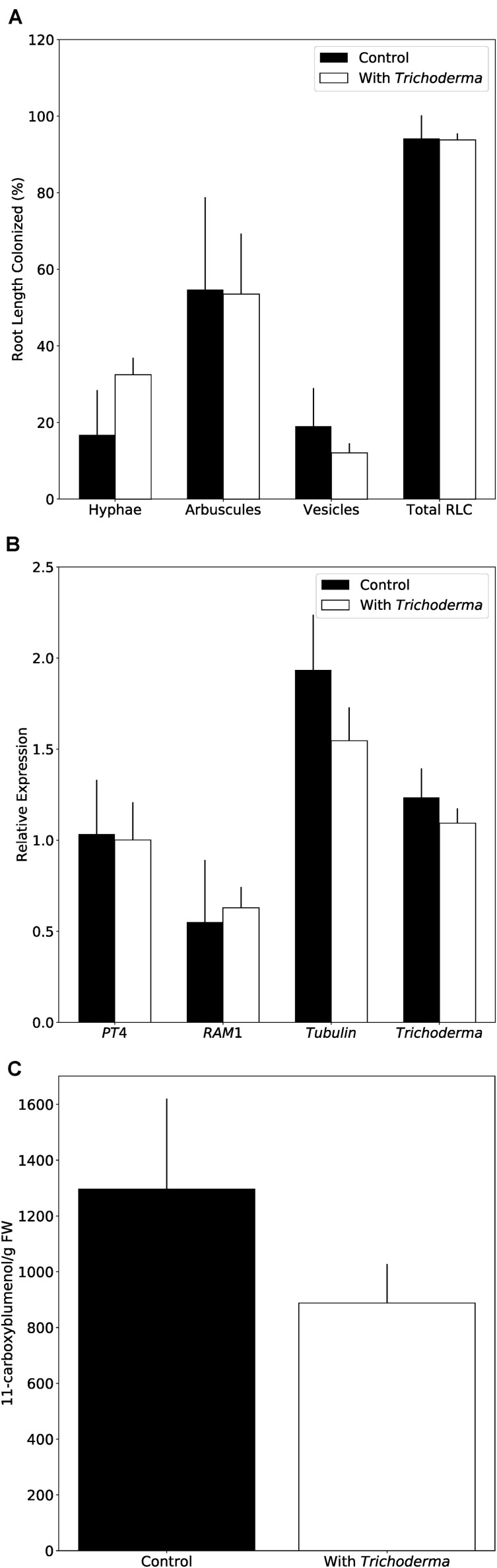
*Trichoderma* does not interfere with mycorrhiza formation on *N. attenuata*. **(A)** AMF colonization 8 weeks after inoculation with or without *Trichoderma* and AMF simultaneously. Error bars represent SEs from three independent biological replicates. Statistical significance was determined by Welch Two Sample *t*-test between control and co-culture treatments and was not significantly different. RLC, Root length colonization. **(B)** AMF marker gene expression determined by qPCR. *PT4*, Phosphate transporter 4; *RAM1*,

Restriction of *Alternaria* growth by the new *Trichoderma* strain indicated a putative use for bioprotection. However, agricultural application requires that other beneficial fungi, such as arbuscular mycorrhizal fungi (AMF) are not affected by the *Trichoderma* strain. As *Arabidopsis* is a non-mycorrhizal species, we used the well-established *N. attenuata* system (Groten et al., 2015). *N. attenuata* plants grown on soil in the greenhouse were simultaneously inoculated with AMF and *Trichoderma.* Microscopic observations of the roots and qPCR analyses with fungus-specific markers clearly indicate that AMF and *Trichoderma* colonize the roots and propagate, without inhibiting each other (Figures 8A,B). In addition, the amounts of 11-carboxyblumenol, a marker for AMF root colonization (Wang et al., 2018a), did not differ between *Trichoderma*-inoculated and non-inoculated samples (Figure 8C). 11-Carboxyblumenol levels were also similar when plants were pre-inoculated with AMF and after 6 weeks co-cultured with *Trichoderma* (data not shown). These results suggest that AMF colonization is not affected by the new *Trichoderma* strain in *N. attenuata*.

### The *Trichoderma* Strain Alters Phytohormone Levels in *Arabidopsis* Roots and Shoots

**FIGURE 9:**
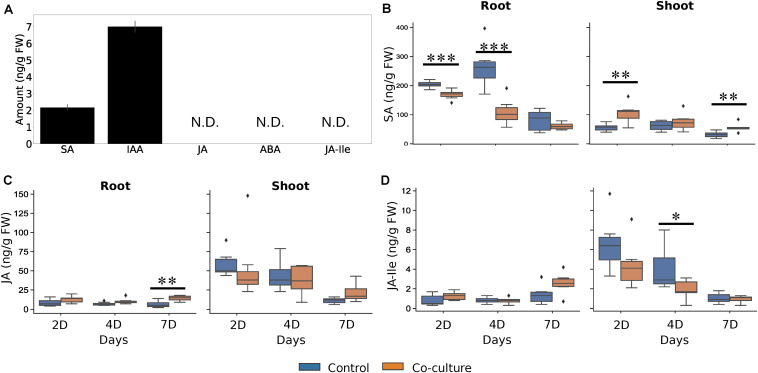
*Trichoderma* facilitates the allocation of phytohormones in *A. thaliana*. **(A)** Phytohormone levels in *Trichoderma* mycelium. Error bars represent the SEs from six independent biological replicates. N.D., not detected. **(B–D)** SA **(B)**, JA **(C)**, and JA-Ile **(D)** levels from control and co-cultured seedlings in roots and shoots 2, 4, and 7 days after co-cultivation. Statistical significance was determined by Welch Two Sample *t*-test between control and co-culture treatments (^∗^*P* < 0.05; ^∗∗^*P* < 0.01; ^∗∗∗^*P* < 0.001). At least 6 biological replicates were used for measurement, each with 16 seedlings. ABA, abscisic acid; IAA, indole-3-acetic acid; SA, salicylic acid; JA, jasmonic acid; JA-Ile, jasmonoyl-isoleucine conjugate. The diamond shape in the figure represent outliers, which the data points exceed 1.5 times of the inter-quartile range from the 75th percentiles, or lower than 1.5 times of the inter-quartile range from the 25th percentiles. The inter-quartile range is the range between 25th and 75th percentiles.

Beneficial plant-microbe interactions often result in altered phytohormone levels, which may lead to better fitness of the host upon pathogen attack but can also influence root colonization due to an altered plant immune system (Jacobs et al., 2011). In mycelial cultures, we detected only trace amounts of auxin (indole-acetic acid, IAA) and SA (Figure 9A). However, SA in *Trichoderma*-colonized seedlings were significantly reduced in roots and increased in shoots compared to controls (Figure 9B). Metabolites related to the biosynthesis and degradation of JA as well as ABA and IAA also showed some minor changes after *Trichoderma* colonization, but compared to SA, these changes were rather weak (Figures 9C,D and Supplementary Figure 6). Overall, it appears that the fungus does not produce high hormone levels itself influencing plant performance, but the fungus may activate SA-dependent resistance responses in the plant.

## Discussion

In this study, a new endophytic *Trichoderma* strain is described. It belongs to the *harzianum* clade, closely related to *T. confertum, T. pleuroti* and *T. pleuroticola*. It survives under salt and osmotic stress, and possesses a strong capability to reduce *A. brassicicola* growth. The hyphae colonize the root surface and are found in root hairs of *A. thaliana*. Infection assays showed reduced *A. brassicicola* spread in roots and shoots of *Trichoderma*-colonized *Arabidopsis* plants, while mycorrhiza formation is not affected in *N. attenuata*. These observations are important for potential application of the endophyte as bio-control agent, and for the development of more effective and versatile bio-control agents.

Numerous *Trichoderma* species have been reported to stimulate plant growth (Contreras-Cornejo et al., 2009; González-Pérez et al., 2018), and *T. atroviride* and *T. virens* have been shown to promote root hair development (Contreras-Cornejo et al., 2015; González-Pérez et al., 2018). Our results highlight the importance of the growth conditions for the investigations of the symbiotic interactions with the new *Trichoderma* strain. Most importantly, as long as the symbionts grow in soil, we observe growth promotion during early phases of the development in the two tested host species. However, the growth stimulating effect of the fungus was barely or not detectable at all on agar plates, as long as no NaCl is added. A possible scenario could be that the fungus requires low concentrations of NaCl for growth and thus root colonization. If the salt concentration in the medium is too high, the fungus helps the plant by stimulating osmolyte production and Na^+^ elimination through root exudates (Contreras-Cornejo et al., 2014). We demonstrate that the fungus also tries to escape from the stress by growing on the plant material, since the roots become more colonized with increased salt concentrations. Ultimately, hyphae can also be detected in the aerial parts of the plant, which occurs only when the stress around the roots is high. We assume that the extensive fungal propagation triggers the plant defense machinery to restrict fungal growth and consequently may reduce the host´s investment into growth. While our experiment focusses on the role of NaCl for the symbiosis, there are apparently other growth-stimulating factors in soil. A comparative analysis of the different growth conditions established in this study may help to elucidate critical parameters with agricultural relevance.

### Root Colonization Alters Root Architecture

The new *Trichoderma* strain not only colonizes the root surface, but also penetrates into the root epidermis and resides in the root hairs (Figures 2E–G and Supplementary Movie 1). To the best of our knowledge, this is a new colonization strategy for *Trichoderma* species and demonstrates that the fungus can also live as endophyte. This finding is further supported by the fact that the fungus was originally isolated from the leaf cells of a tree. Although *Trichoderma* species have been often reported to colonize plant roots (López-Bucio et al., 2015; Ruano-Rosa et al., 2016), the invasion of hyphae into root cells might indicate a closer symbiosis compared to other *Trichoderma* strains and species. Reprogramming of root development, inhibition of root growth and stimulating root branching is a typical feature of AMF (Bonfante and Genre, 2010), but also observed for *Trichoderma*-colonized *Arabidopsis* roots (e.g., Contreras-Cornejo et al., 2015). Similar to AMF associations, the endophyte might contribute to nutrient and water uptake and allow the plants to reduce their root sizes. Further studies are needed to support this hypothesis. Additionally, an increase in the number of root hairs may lead to a larger surface area for fungal attachment. Its close phylogenetic relationship to *Trichoderma* species which grow preferentially on mushrooms also demonstrates that minor changes in the *Trichoderma* genomes allow major changes, enlargements or alterations in their host range or preference.

### The New *Trichoderma* Strain Has Potential as New Bio-Control Agent

The infection assays with *A. brassicicola* show effective protection of *Arabidopsis* roots and shoots by *Trichoderma*. Interestingly, the beneficial fungus also restricted growth of *A. brassicicola* in the roots, when the roots were already infected by the pathogen (Figure 7A, A-T vs. C-T). This is consistent with the plate experiments in which *Trichoderma* actively predated *A. brassicicola*. Propagation of the pathogen in the leaves is also restricted when the roots are colonized by *Trichoderma*. Different local and systemic plant immune responses against various pathogens in *Alternaria*-colonized hosts have been reported, however, a general strategy for *Trichoderma* species is not apparent (Busby et al., 2016; Rai and Agarkar, 2016). Apparently, systemic signals travel from the roots to the leaves, and this is reflected by elevated SA levels in the leaves of *Trichoderma*-colonized seedlings even before they are exposed to the pathogen (Figure 9B). The higher SA levels in the leaves might indicate that the new *Trichoderma* strain has the ability to induce SAR. The low or undetectable levels of the defense-related hormones in the mycelium suggest that they are not of fungal origin.

Another feature of this new strain is its ability to sustain beneficial microbe interaction with plants. Although pathogen progression in root tissue is hindered by the new *Trichoderma* strain, the presence of the fungus does not interfere with AMF colonization. Recently, Metwally and Al-Amri (2020) showed an interactive role of *Trichoderma viride* and AMF on growth and pigment content of onion plants, however, due to the small number of AMF−*Trichoderma*–host plant combinations that have been investigated so far, general conclusions on those tripartite interactions are not possible (cf. Szczałba et al., 2019). Those studies are important for a successful bio-control agent, as *Trichoderma* species are also competitors of beneficial microbes (Sood et al., 2020), which could impair plant growth or yield.

## Conclusion

In conclusion, the new *Trichoderma* strain might be a useful tool as bio-control agent, since it stimulates the plant immune system against pathogen infection, but at the same time does not interfere with other beneficial microbial interactions, such as mycorrhizal formation. Its growth promoting ability in soil provides additional benefit in agricultural application. Furthermore, the experimental set-up allows us to address further questions to understand the role of this fungus on plant performance, especially why the fungus is successful in promoting plant growth in soil but not on minimal medium, and how it influences the balance between growth and stress responses under different environmental conditions.

## Data Availability Statement

The datasets generated for this study can be found in the online repositories. The names of the repository/repositories and accession number(s) can be found in the article/Supplementary Material.

## Author Contributions

Y-HT organized the project, performed the experiments, collected the samples and data, analyzed the results, plotted the figures, and wrote up the study. HR performed the soil and salt experiments on *Arabidopsis*. KG performed the experiments on *Nicotiana*. PR isolated the *Trichoderma* strain. AF assisted in the microscopy. MR measured the phytohormones. IB, KN, RU, and RO edited the manuscript. RO organized the project and wrote up the study. All authors contributed to the article and approved the submitted version.

## Conflict of Interest

The authors declare that the research was conducted in the absence of any commercial or financial relationships that could be construed as a potential conflict of interest.

## Footnotes

1 www.biomyc.de
