# Supplementary material for: An Endophytic Trichoderma Strain Promotes Growth of Its Hosts and Defends Against Pathogen Attack
Source: Front Plant Sci. 2020 Dec 3;11:573670. doi: 10.3389/fpls.2020.573670 (PMC7793846; doi:10.3389/fpls.2020.573670)
Supplement: Supplementary file 1 [file Data_Sheet_1.PDF]

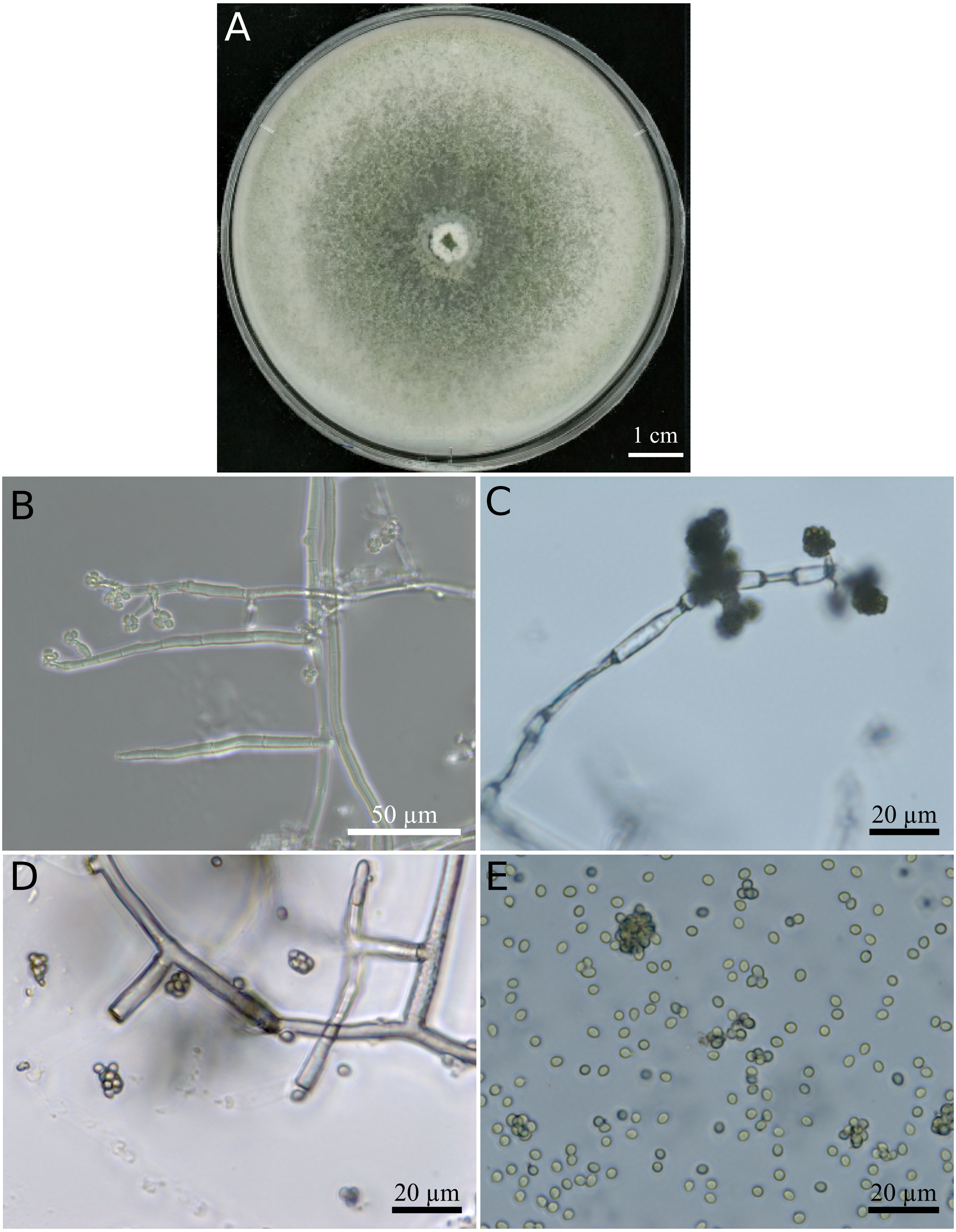

Supplementary Figure 1. Morphology of the new *Trichoderma* strain. **(A)**: The strain on KM plates after 4 days. **(B) - (D)**: Developing conidia 3 **(B)** and 7 **(C - D)** days after incubation. **(E)**: Released conidia.
