# Supplementary material for: An Endophytic Trichoderma Strain Promotes Growth of Its Hosts and Defends Against Pathogen Attack
Source: Front Plant Sci. 2020 Dec 3;11:573670. doi: 10.3389/fpls.2020.573670 (PMC7793846; doi:10.3389/fpls.2020.573670)
Supplement: Supplementary file 2 [file Data_Sheet_2.PDF]

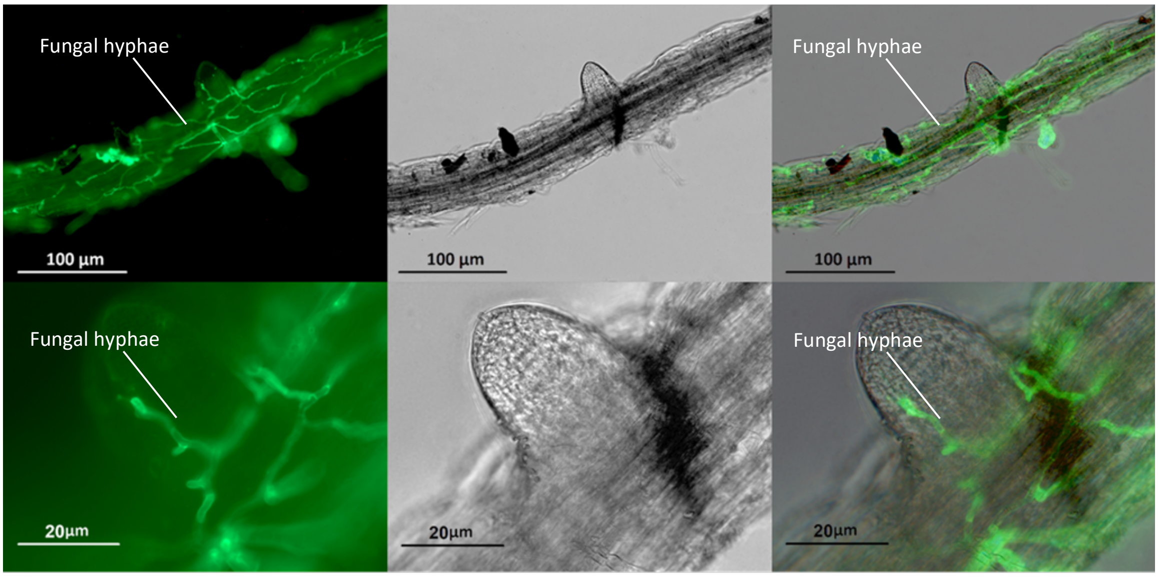

Supplementary Figure 2. Colonization of *Trichoderma* on *Arabidopsis* roots on soil. Left: Fluorescence of fungal stain; Center: bright field; Right: overlay. Fungal hyphae was stained by WGA Alexa Fluor™ 488 conjugate and visualized by fluorescence.
