# Supplementary material for: An Endophytic Trichoderma Strain Promotes Growth of Its Hosts and Defends Against Pathogen Attack
Source: Front Plant Sci. 2020 Dec 3;11:573670. doi: 10.3389/fpls.2020.573670 (PMC7793846; doi:10.3389/fpls.2020.573670)
Supplement: Supplementary file 3 [file Data_Sheet_3.PDF]

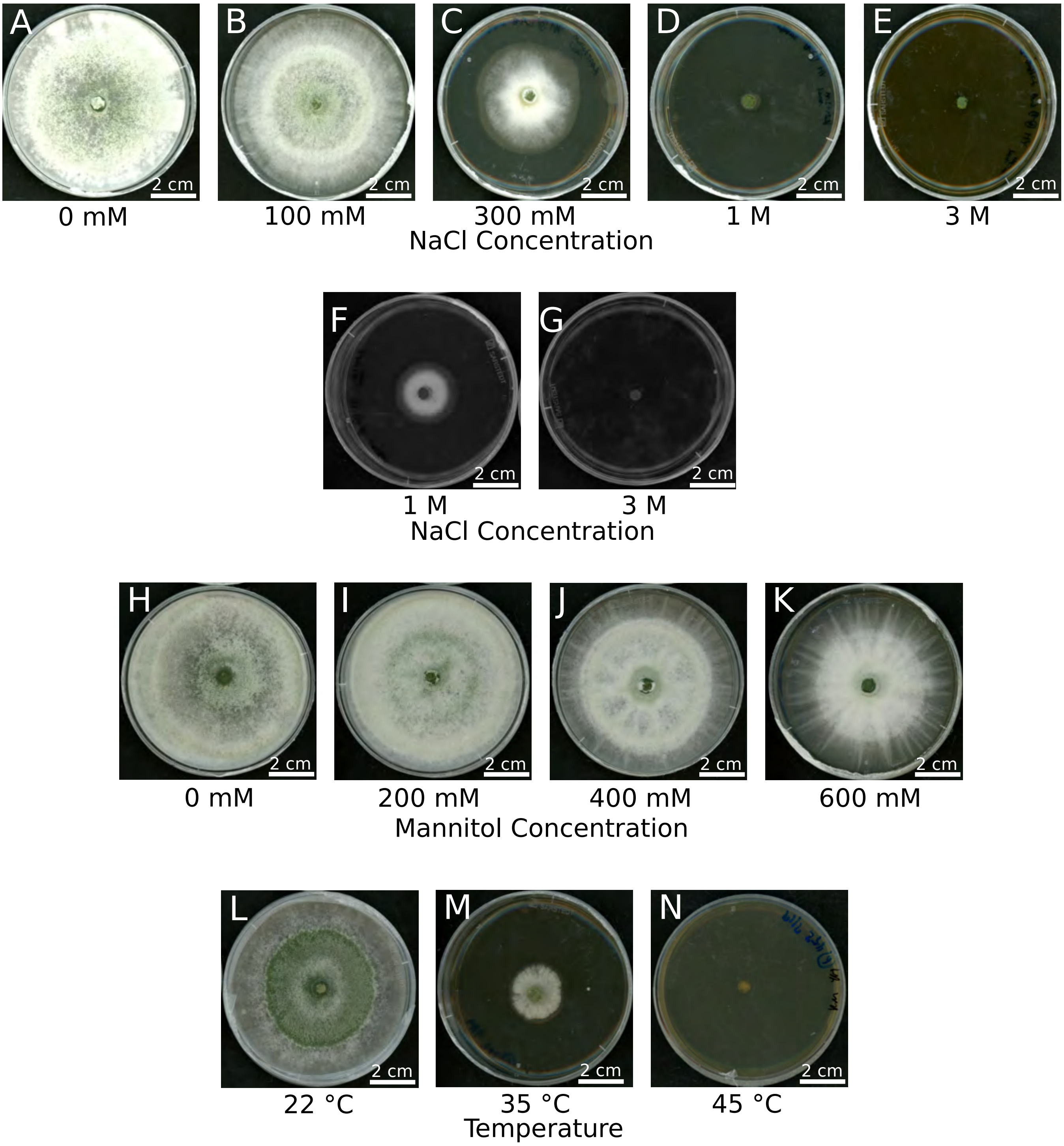


**Supplementary Figure 3**. Growth phenotype of the *Trichoderma* strain under various conditions on KM plates. **(A) - (E)**: Fungal growth on different NaCl concentrations for 4 days. **(F) and (G)**: Mycelial growth on KM plates with 1 M **(F)** or 3 M **(G)** NaCl for 10 days. **(H) - (K)**: Fungal growth on different mannitol concentrations for 4 days. **(L) - (N)**: Fungal growth at different temperatures for 4 days.
