# Supplementary material for: An Endophytic Trichoderma Strain Promotes Growth of Its Hosts and Defends Against Pathogen Attack
Source: Front Plant Sci. 2020 Dec 3;11:573670. doi: 10.3389/fpls.2020.573670 (PMC7793846; doi:10.3389/fpls.2020.573670)
Supplement: Supplementary file 4 [file Data_Sheet_4.PDF]

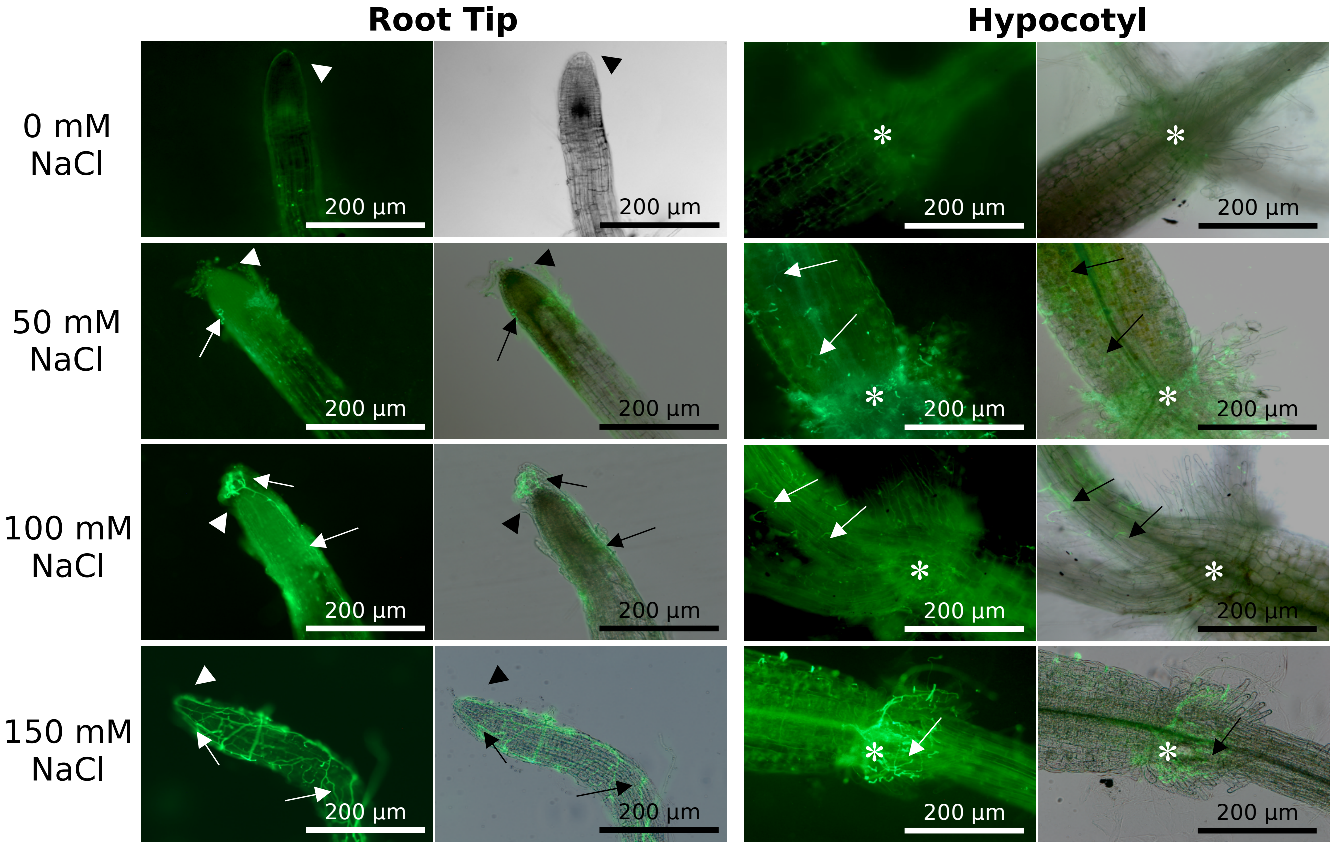

Supplementary Figure 4. Root colonization on *A. thaliana* on PNM medium with 0 - 150 mM NaCl. Root tips, hypocotyls and fungal hyphae are indicated by filled triangles, asterisks and arrows, respectively.
