# Supplementary material for: An Endophytic Trichoderma Strain Promotes Growth of Its Hosts and Defends Against Pathogen Attack
Source: Front Plant Sci. 2020 Dec 3;11:573670. doi: 10.3389/fpls.2020.573670 (PMC7793846; doi:10.3389/fpls.2020.573670)
Supplement: Supplementary file 5 [file Data_Sheet_5.PDF]

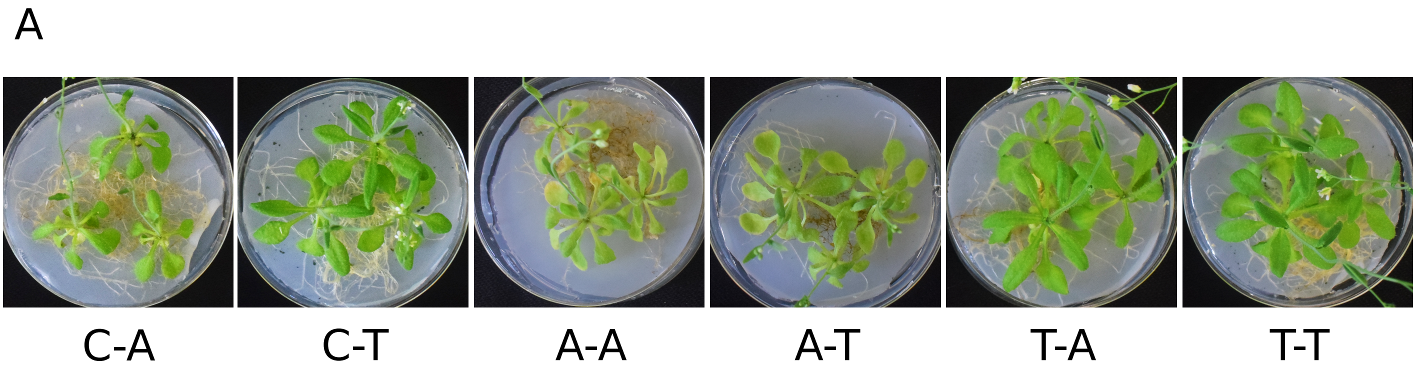

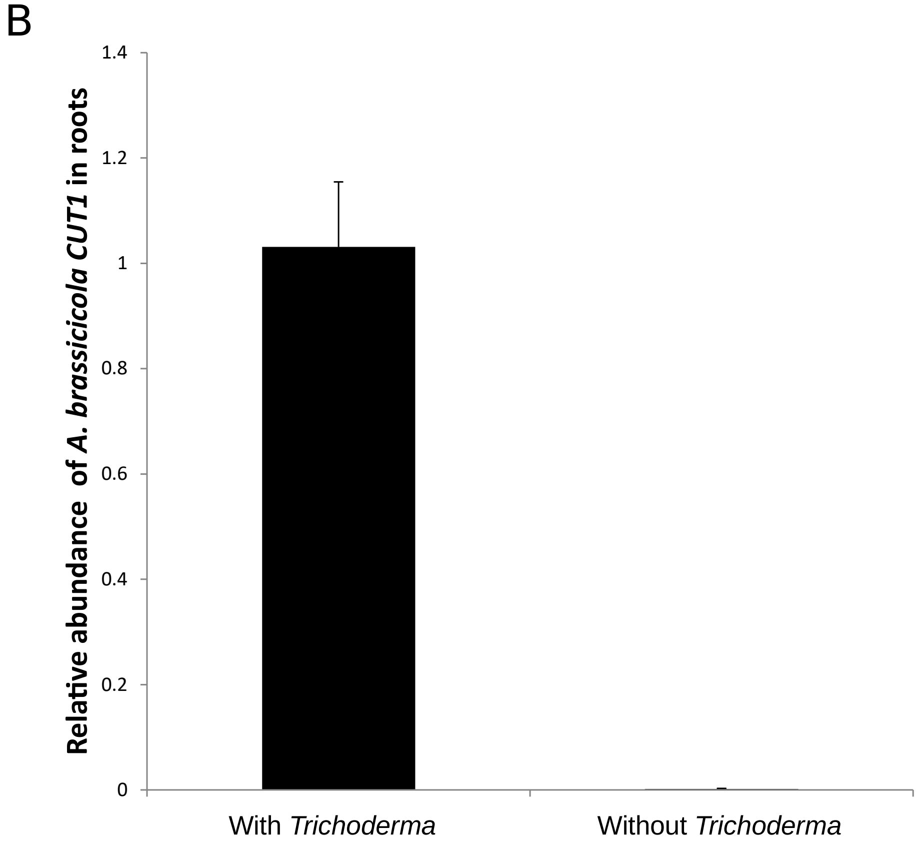

Supplementary Figure 5. *Trichoderma* strain prevents *A. brassicicola* spread in root. **(A)**: Growth of *Arabidopsis* seedlings infected by *A. brassiciciola* or cultivated with the *Trichoderma* strain. C-A: co-cultivation with control plug for 7 days, then co-cultivation with *A. brassicicola* for another 7 days. C-T: co-cultivation with control plug for 7 days, then co-cultivation with *Trichoderma* for another 7 days. A-A: co-cultivation with *A. brassicicola* for 7 days, then co-cultivation with *A. brassicicola* for another 7 days. A-T: co-cultivation with *A. brassicicola* for 7 days, then co-cultivation with *Trichoderma* for another 7 days. T-A: co-cultivation with *Trichoderma* for 7 days, then co-cultivation with *A. brassicicola* for another 7 days. T-T: co-cultivation with *Trichoderma* for 7 days, then co-cultivation with *Trichoderma* for another 7 days. **(B)**: Relative DNA Amount of *A. brassicicola CUT1* to *N. attenuata TEF1*.
