# Supplementary material for: An Endophytic Trichoderma Strain Promotes Growth of Its Hosts and Defends Against Pathogen Attack
Source: Front Plant Sci. 2020 Dec 3;11:573670. doi: 10.3389/fpls.2020.573670 (PMC7793846; doi:10.3389/fpls.2020.573670)
Supplement: Supplementary file 6 [file Data_Sheet_6.PDF]

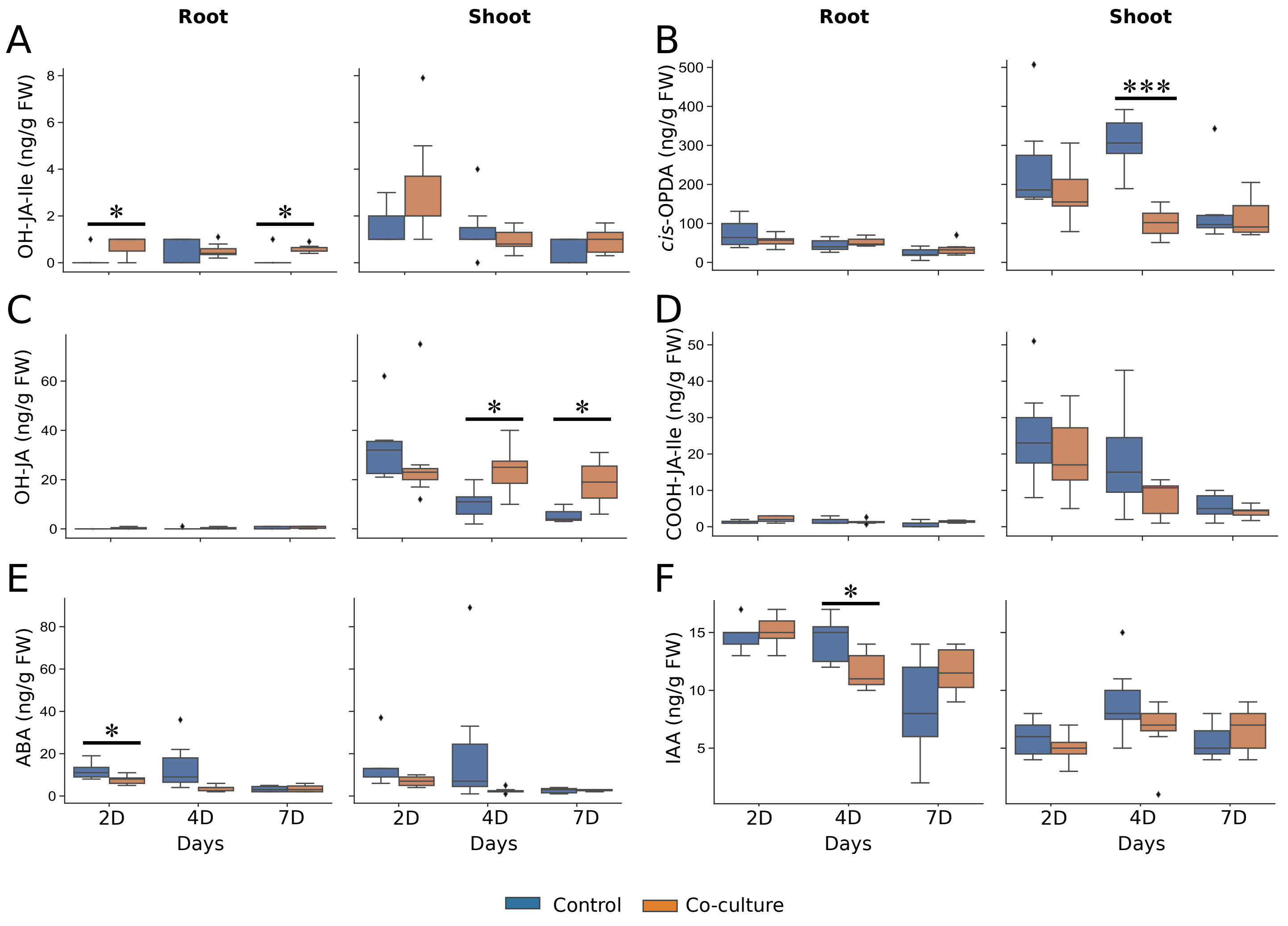

Supplementary Figure 6. Phytohormone redistribution in *A. thaliana* facilitated by *Trichoderma*. **(A)**: OH-JA-Ile **(B)**: *cis*-OPDA **(C)**: OH-JA **(D)**: COOH-JA-Ile **(E)**: ABA and **(F)**: IAA levels from control and co-cultured seedlings in roots and shoots 2, 4 and 7 days after co-cultivation. Statistical significance was determined by Welch Two Sample t-test between control and co-culture treatments (*$P < 0.05$; ***$P < 0.001$). At least 6 biological replicates were used for measurement, each with 16 seedlings. ABA: abscisic acid; IAA: indole-3-acetic acid; *cis*-OPDA: *cis*-12-oxo-phytodienoic acid; OH-JA: 12-hydroxyl jasmonic acid; OH-JA-Ile: 12-hydroxyl-jasmonyl-isoleucine; COOH-JA-Ile: 12-carboxyl-jasmonyl-isoleucine. The diamond shape in the figure represent outliers, which the data points exceed 1.5 times of the inter-quartile range from the 75th percentiles, or lower than 1.5 times of the inter-quartile range from the 25th percentiles. The inter-quartile range is the range between 25th and 75th percentiles.
