# Supplementary material for: An Endophytic Trichoderma Strain Promotes Growth of Its Hosts and Defends Against Pathogen Attack
Source: Front Plant Sci. 2020 Dec 3;11:573670. doi: 10.3389/fpls.2020.573670 (PMC7793846; doi:10.3389/fpls.2020.573670)
Supplement: Supplementary file 7 [file Table_1.DOCX]

**Supplementary Table 1.** Sequence accessions used in the multilocus phylogenetic analysis.

| Name | Strain | GenBank Accession Number | |
| --- | --- | --- | --- |
|  |  | *TEF1* | *RPB2* |
| *Trichoderma* sp. in this study |  | MT591352 | MT602550 |
| *T. pleuroti* | CBS 124387 | HM142382 | HM142372 |
| *T. pleuroticola* | CBS 124383 | HM142381 | HM142371 |
| *T. afroharzianum* | GJS 04-186 | FJ463301 | FJ442691 |
| *T. afarasin* | Dis341F | FJ463400 | FJ442778 |
| *T. asperelloides* | GJS 04-187 | JN133571 | JN133560 |
|  | GJS 04-116 | GU248412 | GU248411 |
| *T.asperellum* | CBS 433.97 | AY376058.1 | EU248617.1 |
|  | GJS 90-7 | EU338333 | EU338337 |
|  | GJS 01-294 | EU856323 | FJ150788 |
|  | GJS 06-294 | GU198235 | GU198266 |
|  | CGMCC 6422 | KF425756 | KF425755 |
|  | GJS 05-328 | EU248627 | EU248614 |
| *T. atrobrunneum* | GJS 04-67 | FJ463360 | FJ442724 |
| *T. atroviride* | DAOM 222144 | AF456889 | FJ442754 |
| *T. gamsii* | GJS 04-09 | DQ307541 | JN133561 |
| *T. harzianum* | T55 | KX632625 | KX632568 |
|  | T18 | KX632606 | KX632549 |
|  | T2 | KX632591 | KX632534 |
|  | CBS 226.95 | AF348101 | AF545549 |
|  | T11 | KX632486 | KX632543 |
| *T. hispanicum* | S453 | JN715659 | JN715600 |
| *T. inhamatum* | CBS 273.78 | AF348099 | FJ442725 |
| *T. junci* | CBS 120926 | FJ860641 | FJ860540 |
| *T. lentiforme* | Dis 218E | FJ463310 | FJ442793 |
| *T. lieckfeldtiae* | GJS 00-14 | EU856326 | EU883562 |
| *T. lixii* | GJS 97-96 | AF443938 | KJ665290 |
| *T. rifaii* | Dis 337F | FJ463321 | FJ442720 |
| *T. samuelsii* | S5 | JN715651 | JN715599 |
| *T. viride* | CBS 119325 | DQ672615 | EU711362 |
| *T. longibrachiatum* | CBS 816.68 | AY865640.1 | DQ087242.1 |
| *T. confertum* | TC62 | MF371218 | MF371203 |
|  | TC139 | MF371220 | MF371205 |
| *T. aggressivum* | CBS 100525 | AF534614 | AF545541 |
| *T. alni* | CBS 120633 | EU498312 | EU498349 |
| *T. amazonicum* | IB 95 | HM142377 | HM142368 |
| *T. atrogelatinosum* | LU 498 | KJ871087 | KJ842176 |
| *T. velutinum* | CPK 298 | KJ665769 | KF134794 |
| *T. tomentosum* | DAOM 178713a | AF534630 | AF545557 |
| *T. tawa* | GJS 97-174 | AY392004 | AY391956 |
| *T. stramineum* | GJS 02-84 | AY391999 | AY391945 |
| *T. rufobrunneum* | HMAS 266614 | KF729989 | KF730010 |
| *T. catoptron* | GJS 02-76 | AY391963 | AY391900 |
| *T. pseudogelatinosum* | CNUN 309 | HM920202 | HM920173 |
| *T. priscilae* | S168 | KJ665691 | KJ665333 |
| *T. parepimyces* | CBS 122769 | FJ860664 | FJ860562 |
| *T. longifialidicum* | LESF 552 | KT279020 | KT278955 |
| *T. ceraceum* | GJS 88-28 | AY391964 | AY391901 |
| *T. cerinum* | DAOM 230012 | AY605802 | KJ842184 |
| *T. christiani* | S442 | KJ665439 | KJ665244 |
| *T. cinnamomeum* | GJS 97-237 | AY391979 | AY391920 |
| *T. compactum* | CBS 121218 | KF134798 | KF134789 |
| *T. corneum* | GJS 97-82 | KJ665455 | KJ665252 |
| *T. dacrymycellum* | WU 29044 | FJ860633 | FJ860533 |
| *T. epimyces* | CBS 120534 | EU498320 | EU498360 |
| *T. italicum* | S131 | KJ665525 | KJ665282 |
| *Nectria eustromatica* | CBS 125578 | HM534876 | HM534887 |
