# Supplementary material for: An Endophytic Trichoderma Strain Promotes Growth of Its Hosts and Defends Against Pathogen Attack
Source: Front Plant Sci. 2020 Dec 3;11:573670. doi: 10.3389/fpls.2020.573670 (PMC7793846; doi:10.3389/fpls.2020.573670)
Supplement: Supplementary file 8 [file Table_2.DOCX]

**Supplementary Table 2.** Details of phytohormone analysis by LC-MS/MS [HPLC 1260 (Agilent Technologies)-QTRAP6500 (SCIEX)] in negative ionization mode.

| **Q1** | **Q3** | **RT (min)** | **Compound** | **Internal standard** | **RF** | **DP** | **EP** | **CE** | **CXP** |
| --- | --- | --- | --- | --- | --- | --- | --- | --- | --- |
| 136.93 | 93 | 3.3 | SA | D4-SA | 1.0 | -20 | -8 | -24 | -7 |
| 263 | 153.2 | 3.4 | ABA | D6-ABA | 1.0 | -20 | -12 | -22 | -2 |
| 209.07 | 59 | 3.6 | JA | D6-JA | 1.0 | -20 | -9 | -24 | -2 |
| 322.19 | 130.1 | 3.9 | JA-Ile | D6-JA-Ile | 1.0 | -50 | -4.5 | -30 | -4 |
| 290.9 | 165.1 | 4.6 | OPDA | D6-JA | 1.0 | -20 | -12 | -24 | -2 |
| 338.1 | 130.1 | 3 | OH-JA-Ile | D6-JA-Ile | 1.0 | -50 | -4.5 | -30 | -4 |
| 225.1 | 59 | 2.6 | OH-JA | D6-JA | 1.0 | -20 | -9 | -24 | -2 |
| 352.1 | 130.1 | 3 | COOH-JA-Ile | D6-JA-Ile | 1.0 | -50 | -4.5 | -30 | -4 |
| 140.93 | 97 | 3.3 | D4-SA |  |  | -20 | -8 | -24 | -7 |
| 269 | 159.2 | 3.4 | D6-ABA |  |  | -20 | -12 | -22 | -2 |
| 215 | 59 | 3.6 | D6-JA |  |  | -20 | -9 | -24 | -2 |
| 214 | 59 | 3.6 | D5-JA |  |  | -20 | -9 | -24 | -2 |
| 328.19 | 130.1 | 3.9 | D6-JA-Ile |  |  | -50 | -4.5 | -30 | -4 |
| 327.19 | 130.1 | 3.9 | D5-JA-Ile |  |  | -50 | -4.5 | -30 | -4 |
