# Supplementary material for: An Endophytic Trichoderma Strain Promotes Growth of Its Hosts and Defends Against Pathogen Attack
Source: Front Plant Sci. 2020 Dec 3;11:573670. doi: 10.3389/fpls.2020.573670 (PMC7793846; doi:10.3389/fpls.2020.573670)
Supplement: Supplementary file 9 [file Table_3.DOCX]

**Supplementary Table 3**. Post-hoc analysis of *Arabidopsis* fresh/dry weight with (Y) or without (N) *Trichoderma* under different salt concentrations by Tukey HSD.

| Fresh Weight | Difference (mg) | p adj. |
| --- | --- | --- |
| Y:0mM-N:0mM | 83.3333 | 0.3986 |
| N:50mM-N:0mM | -66.6667 | 0.6509 |
| Y:50mM-N:0mM | 613.3333 | 0.0000 |
| N:100mM-N:0mM | -116.6667 | 0.0997 |
| Y:100mM-N:0mM | -136.6667 | 0.0376 |
| N:150mM-N:0mM | -70.0000 | 0.5986 |
| Y:150mM-N:0mM | -60.0000 | 0.7516 |
| N:50mM-Y:0mM | -150.0000 | 0.0191 |
| Y:50mM-Y:0mM | 530.0000 | 0.0000 |
| N:100mM-Y:0mM | -200.0000 | 0.0015 |
| Y:100mM-Y:0mM | -220.0000 | 0.0005 |
| N:150mM-Y:0mM | -153.3333 | 0.0161 |
| Y:150mM-Y:0mM | -143.3333 | 0.0269 |
| Y:50mM-N:50mM | 680.0000 | 0.0000 |
| N:100mM-N:50mM | -50.0000 | 0.8776 |
| Y:100mM-N:50mM | -70.0000 | 0.5986 |
| N:150mM-N:50mM | -3.3333 | 1.0000 |
| Y:150mM-N:50mM | 6.6667 | 1.0000 |
| N:100mM-Y:50mM | -730.0000 | 0.0000 |
| Y:100mM-Y:50mM | -750.0000 | 0.0000 |
| N:150mM-Y:50mM | -683.3333 | 0.0000 |
| Y:150mM-Y:50mM | -673.3333 | 0.0000 |
| Y:100mM-N:100mM | -20.0000 | 0.9993 |
| N:150mM-N:100mM | 46.6667 | 0.9097 |
| Y:150mM-N:100mM | 56.6667 | 0.7979 |
| N:150mM-Y:100mM | 66.6667 | 0.6509 |
| Y:150mM-Y:100mM | 76.6667 | 0.4952 |
| Y:150mM-N:150mM | 10.0000 | 1.0000 |
| Dry Weight | Difference (mg) | p adj. |
| Y:0mM-N:0mM | 26.6667 | 0.7569 |
| N:50mM-N:0mM | -43.3333 | 0.2387 |
| Y:50mM-N:0mM | 96.6667 | 0.0007 |
| N:100mM-N:0mM | -53.3333 | 0.0882 |
| Y:100mM-N:0mM | -43.3333 | 0.2387 |
| N:150mM-N:0mM | -30.0000 | 0.6444 |
| Y:150mM-N:0mM | -6.6667 | 0.9999 |
| N:50mM-Y:0mM | -70.0000 | 0.0137 |
| Y:50mM-Y:0mM | 70.0000 | 0.0137 |
| N:100mM-Y:0mM | -80.0000 | 0.0043 |
| Y:100mM-Y:0mM | -70.0000 | 0.0137 |
| N:150mM-Y:0mM | -56.6667 | 0.0616 |
| Y:150mM-Y:0mM | -33.3333 | 0.5278 |
| Y:50mM-N:50mM | 140.0000 | 0.0000 |
| N:100mM-N:50mM | -10.0000 | 0.9985 |
| Y:100mM-N:50mM | 0.0000 | 1.0000 |
| N:150mM-N:50mM | 13.3333 | 0.9914 |
| Y:150mM-N:50mM | 36.6667 | 0.4173 |
| N:100mM-Y:50mM | -150.0000 | 0.0000 |
| Y:100mM-Y:50mM | -140.0000 | 0.0000 |
| N:150mM-Y:50mM | -126.6667 | 0.0000 |
| Y:150mM-Y:50mM | -103.3333 | 0.0003 |
| Y:100mM-N:100mM | 10.0000 | 0.9985 |
| N:150mM-N:100mM | 23.3333 | 0.8538 |
| Y:150mM-N:100mM | 46.6667 | 0.1743 |
| N:150mM-Y:100mM | 13.3333 | 0.9914 |
| Y:150mM-Y:100mM | 36.6667 | 0.4173 |
| Y:150mM-N:150mM | 23.3333 | 0.8538 |
